# Effects of sampling site, season, and substrate on foraminiferal assemblages grown from propagule banks from lagoon sediments of Corfu Island (Greece, Ionian Sea)

**DOI:** 10.1371/journal.pone.0219015

**Published:** 2019-06-28

**Authors:** Anna E. Weinmann, Susan T. Goldstein, Maria V. Triantaphyllou, Martin R. Langer

**Affiliations:** 1 Institut für Geowissenschaften und Meteorologie, Abteilung Paläontologie, Rheinische Friedrich-Wilhelms-Universität Bonn, Bonn, Germany; 2 Department of Geology, University of Georgia, Athens, Georgia, United States of America; 3 Faculty of Geology and Geoenvironment, National and Kapodistrian University of Athens, Athens, Greece; Universita degli Studi di Urbino Carlo Bo, ITALY

## Abstract

Foraminiferal propagule banks occur in fine sediment fractions that contain small individuals of benthic foraminifera. These sediments include locally sourced juveniles and propagules, as well as allochthonous propagules that have dispersed from surrounding areas. Such propagules can remain viable even under unfavorable local conditions. When exposed to more favorable conditions, they may grow to adult stages. Accordingly, during environmental changes, propagule banks have the potential to function as species pools and allow quick assemblage reactions. The propagule method was designed to study responses of foraminiferal assemblages by exposing propagule banks to controlled conditions in the laboratory, an approach that is applicable to a variety of ecological questions. Therefore it is important to understand the nature and dynamics of propagule banks, including local and seasonal influences. To obtain insights into the composition of local propagule banks, we studied experimentally grown assemblages from two shallow-water lagoons on Corfu Island in western Greece, and compared the results with in situ assemblages. We sampled in spring and autumn of 2017 and experimental treatments included the use of different substrates in our experiments to account for potential effects on assemblage compositions. Results revealed that sediments from each lagoon contained a distinct propagule bank. We found abundant allochthonous taxa among specimens grown in all experimental treatments, indicating dispersal of propagules, and possibly also juveniles, from adjacent regions into both lagoons. The time of sampling had a significant effect on experimental assemblages, indicating that the composition of propagule banks can vary throughout the year. However, no significant differences were found in assemblages grown in different substrata, suggesting a stronger influence of water variables (e.g., temperature or salinity) on assemblage compositions. Moreover, the experimental set-ups favored small, fast-growing, sediment-dwelling species tolerant of relatively high organic content. Our findings highlight the potential of propagule banks as species pools and will help to refine and improve future applications of the method.

## Introduction

Assemblages of benthic foraminifera are widespread and nearly ubiquitous in the modern oceans. In shallow-water, coastal environments they are found in almost all habitats and form habitat-specific assemblages, depending on environmental conditions and available microhabitats (e.g., [1, 2]). As such, they are widely applicable in ecological and paleoecological research and are increasingly used as indicators for bio-monitoring [2–9]. Most studies focus on adult foraminiferal assemblages.

The fine size fraction of the sediment, however, contains numerous living small juvenile forms and cryptobiotic propagules. Such specimens, which can either derive from reproduction of local populations or be transported to the respective sites from adjacent areas, form the so-called propagule bank. The term “propagule” refers to tiny juveniles (maybe just the proloculus [10]) that can easily be dispersed due to their small size and be transported well beyond where they were produced [10–12]. A variety of dispersal mechanisms are known for benthic foraminifera including transport in suspension by currents [13, 14], rafting on objects (e.g., [15]) or carried in the intestinal tracts of metazoans, including fish [16] or on feet and feathers of birds [17, 18]. Thus, dispersal in form of propagules appears to be quite widespread among many species of benthic foraminifera [11, 19, 20]. If propagules are transported into habitats outside their normal environmental conditions, they can become dormant [21], which has also been observed in adult foraminifera [22], and can remain viable within the local propagule bank for at least two years [19]. If local environmental conditions should become suitable (e.g., due to climate change), subsequent growth of the propagules to adult populations is possible [10, 19]. Recent observations in the Bottsand lagoon (Baltic Sea) have provided field evidence of the presence of allochthonous propagules, as the previously absent *Elphidium incertum* suddenly appeared among local assemblages after a short period of increased salinity and even prevailed after conditions returned to their original state [23].

The propagule method is an experimental procedure in which propagule banks are concentrated in the fine fraction by removing the coarser sediments (e.g., using sediment sieves). Subsamples of the fine sediments are then placed under different environmental conditions (i.e., experimental treatments) in the laboratory [11, 12]. The exposure to “new” conditions can lead to growth and even reproduction of faunal assemblages that differ in diversity and faunal composition from the in situ assemblage at the respective collection site [10, 12, 19, 20, 24]. Allochthonous species have been documented in several growth experiments using the propagule method (e.g., [21, 25, 26]). Some studies have recorded shallow-water species that grew from sediments collected from deep-water environments [10, 19]. Others documented the occurrence of marsh or open-shelf species in experimental assemblages from shallow-water or intertidal study sites [12, 20, 24].

The presence of allochthonous species within the propagule banks, which are not normally observed within the in situ assemblages (>63 μm), significantly increases the overall diversity of foraminiferal communities in various environments (e.g., [19, 24]). As such, they can contribute to community structures in the sense of a “species pool”, a hypothesis introduced by Buzas and Culver [27]. It postulates that any community at a certain time can be considered as a subset of a larger species pool that includes all potential immigrants and emigrants from adjacent habitats over time, as long as these habitats are connected [27]. This explains rapid faunal responses in benthic foraminiferal assemblages that were previously observed (e.g., [28]). The hidden diversity of the propagule banks can contribute to such responses, since dormant propagules might be present at a site at any time. In the wake of ongoing global ecological alterations to aquatic environments globally, it becomes more important to analyze and quantify community structures and dynamics for potential response mechanisms, including local propagule banks.

The Mediterranean Sea is a highly suitable area for such studies, because it is one of the most severely impacted marine regions with regard to ongoing environmental changes (e.g., [29]). Climate change leads to increasing temperatures and sea-level rise. The latter will strongly affect shallow-water environments such as coastal lagoons, which face the possibilities of a continuous “marinization” [30]. Coastal lagoons, including those in Greek waters, harbor specific foraminiferal communities (e.g., [31–39]), which are modified as a result of ecological alterations.

Under conditions of global environmental change, the role of propagule banks as potential species pool contributors may become even more important. As such, an important research target is the analysis of structures and dynamics of those propagule banks. A better knowledge and understanding of propagule banks may improve and refine future growth experiments using the propagule method, which provides a wide range of potential applications [11].

To enhance our understanding of the composition and dynamics of local propagule banks, we conducted growth experiments on material from two shallow-water lagoons on the eastern coast of Corfu Island (Western Greece): Chalikiopoulou and Antinioti. Our experimental set-ups were designed to target the following research questions:

1) How does the sampling site influence the faunal composition of local propagule banks? Previous studies have already demonstrated the presence of individually composed propagule assemblages at sites that differ environmentally (e.g., with regard to temperature and salinity [20, 24]). Yet differences between sites that are ecologically similar have not been documented. Furthermore, adult foraminiferal assemblages in shallow-marine environments often exhibit patchy distributions [40–42] and it has not yet been determined if propagule banks are also patchy.

2) How does the time of sampling influence the composition of local propagule banks? A comparison between two previous growth experiments using propagule banks from sediments that were sampled at the same site but at different times of the year, showed noticeable differences in their respective experimental assemblages, even under comparable experimental conditions [12, 20]. As shallow-water benthic foraminifera exhibit high variability in reproduction cycles (e.g., [41, 43–45]), this should lead to seasonal variations in propagule production and subsequent dispersal and settling.

3) How do different types of substrate influence the composition of assemblages grown from the same propagule banks? Since shallow-water foraminiferal species exhibit a large variety of life-modes (e.g., epiphytic, infaunal, etc.) [2], the availability of offered additional microhabitats during the experiments may affect the resulting assemblage compositions.

## Material and methods

### Sampling area

**Fig 1:**
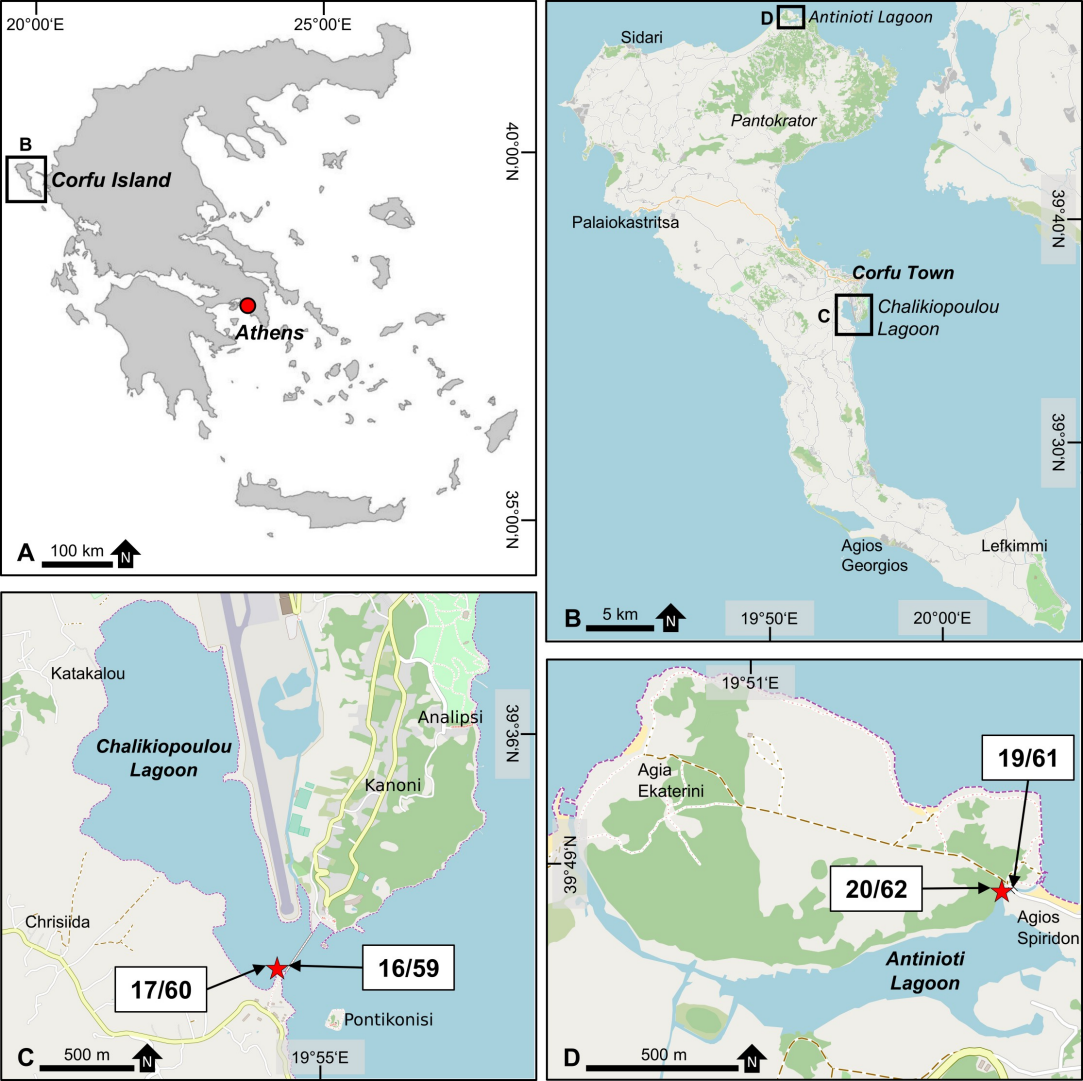
Location maps of the sampling sites. (A) Overview map with location of Corfu Island. Base map modified from GingkoMaps (http://www.ginkgomaps.com/) (B) Overview map of Corfu Island with location of the two lagoons. (C) Chalikiopoulou lagoon with location of two subsamples taken in May and October 2017 (see also Table 1). (D) Antinioti lagoon with location of two subsamples taken in May and October 2017 (see also Table 1). B-D base maps modified from OpenStreetMap (www.openstreetmap.org).

Sediment samples were taken from two shallow-water lagoons at Corfu Island: Chalikiopoulou lagoon on the eastern coast and Antinioti lagoon on the northeastern coast (Fig 1). Sampling was permitted through collaborative research with the National and Kapodistrian University Athens. No additional specific permissions were required for this work, which also does not involve endangered or protected species. Both lagoons exhibited calm and sheltered conditions for the accumulation of fine sediments, which is important for the collection of propagule banks. At the same time, both lagoons were linked to the open ocean, featuring a high connectivity with surrounding habitats.

Chalikiopoulou lagoon is situated south of Corfu Town. To the east, it is separated from the sea by the Kanoni Peninsula, leaving only a small inlet (approximately 300 m) to the south. The lagoon has a north-south extension of 2.1 km and a maximum east-west extension of 1.4 km. On its eastern coast, it is crossed by the runway of Corfu Airport. Nevertheless, the ecological status of the lagoon is good and it is a Natura 2000 site (http://ec.europa.eu/environment/nature/natura2000/). Local fishing activities are common. The average water depth of the lagoon is <1 m [46] and tidal influence is low with a range of ~20 cm (personal observation and www.tide-forecast.com). The inlet is bridged by a narrow wall with several larger openings to allow tidal currents to flow in and out of the lagoon. Near the inlet, a continuous current is visible. The sediment at the sampling site near the inlet is greyish in color and is composed of mostly silt, mud, and fine sand. Bioturbation is common. Organic material (mostly plant debris from algae and surrounding vegetation) is visible, and the sediments turn black below 1 cm of sediment depth.

Antinioti lagoon is located on the northernmost point of the island, approximately 25 km north of Corfu Town. The main lagoon has an east-west extension of 1 km and a north-south extension of 270 m. It is connected to the open ocean by an elongated inlet to the northwest and a shorter inlet to the east. Water depth is comparable to Chalikiopoulou lagoon and the tidal range is only a few centimeters (personal observation and www.tide-forecast.com). The lagoon is used for fish and shrimp farming and is also a Natura 2000 site. Unlike Chalikiopoulou, no current is visible ~100 m behind the inlet mouth and the water is very calm. The sediment at the sampling site near the eastern inlet is brown-grey in color and is composed of silt, mud, and very fine sand. Especially in the still-water areas behind small shrubs of vegetation, organic material (mostly plant debris) is clearly visible and the sediment turns dark after a few millimeters. Bioturbation is less distinct compared to Chalikiopoulou lagoon.

### Sample collection and treatment

Samples were taken at both sampling sites in spring (May 26) and autumn of 2017 (October 13) from mudbanks that were partially exposed during low tide. Water temperature and salinity values measured onsite with a digital thermometer (Extech Instruments, 0.1°C resolution, ±1°C accuracy) and a hand-held seawater refractometer (Red Sea) are provided in Table 1. Samples were taken by scraping the upper-most sediment layers (max. 5 mm) within two areas of approximately 5 m^2^ per lagoon, resulting in two subsamples of ~1–2 L per site. The material was placed into 5 L plastic containers and carefully homogenized.

**Table 1:** Details of sample stations on Corfu Island.

Sample no.	Sampling site	Sampling date	Latitude	Longitude	Water temp. [°C]	Salinity [ppt]
**16**	Chalikiopoulou 1	26/05/2017	39°35’19.61”	19°54’52.42”	20.5	39
**17**	Chalikiopoulou 2	26/05/2017	39°35’19.81”	19°54’50.78”	20.5	39
**59**	Chalikiopoulou 1	13/10/2017	39°35’19.61”	19°54’52.42”	19.2	40.5
**60**	Chalikiopoulou 2	13/10/2017	39°35’19.81”	19°54’50.78”	22.6	40.5
**19**	Antinioti 1	26/05/2017	39°48’57.81”	19°51’33.25	21.9	40
**20**	Antinioti 2	26/05/2017	39°48’57.73”	19°51’33.02”	21.9	40
**61**	Antinioti 1	13/10/2017	39°48’57.81”	19°51’33.25	24.0	25
**62**	Antinioti 2	13/10/2017	39°48’57.73”	19°51’33.02”	24.0	25

Water temperature and salinity were measured at the time of sampling.

After collection, the sediments were sieved on site using ambient seawater and a 53-μm stainless-steel sieve (as in [12, 20, 24]). The fractions >53 μm were preserved in 90% ethanol (buffered with sodium carbonate to avoid acidic conditions) with 2 grams of rose Bengal per liter of ethanol to distinguish the living and dead assemblages (modified from [47]). Despite some challenges, the staining with rose Bengal is deemed a sufficiently reliable technique in warm and oxygenated environments [48] and can serve as an estimate of the living assemblage. After two weeks, the stained samples were washed over a 63-μm sieve and dried for at least 48h at 50°C. Foraminifera from each sample (see Table 1) were picked (until ~300 benthic specimens), identified, and counted (S1 Table). Species identification was mainly based on [49–55].

The fractions <53 μm were collected in 5 L plastic containers. After the suspended sediment settled, the subsamples contained approximately 100–200 ml of fine sediment. On May 27 and October 14 2017, the containers were sealed with parafilm and placed in insulated boxes for transport. A temperature logger (LOG200, Dostmann electronic) was placed within one of the boxes for the monitoring of ambient temperature during the 18h transport. In May, temperatures during transport varied between 16–22.8°C and in October they ranged from 17.8–22.6°C. After arrival at the micropaleontological laboratory of the University of Bonn (Germany), the containers were opened and the suspension was left to settle for approximately 28h before being processed for the growth experiments. Temperatures in the laboratory ranged from 21.5–26.3°C in May and from 19.7–22.5°C in October. Experiments commenced on May 30 and October 17, 2017, respectively.

### Growth experiments

Growth experiments were performed using the Propagule Method [11, 12] with some modifications to adjust for the local conditions of the sampling sites and the research questions. From the fine fraction (<53 μm), 10-ml subsamples of sediment were taken and placed into translucent containers (100 ml; polypropylene), along with 60 ml of artificial seawater (Coral Pro salt, Red Sea). The concentration of the Coral Pro was adjusted to a salinity of 40 ppt, which was close to ambient conditions, except for Antinioti in October (see Table 1). Salinity values within the containers were checked twice per week with a hand-held seawater refractometer (Red Sea). To simulate different additional substrata, 3 leaves of an artificial water plant and 15 pieces of rubble-sized hydrocarbonate (Aqua Medic, size fraction 5 mm to 1 cm) were placed into one third of the containers respectively while the remaining third was left as plain mud sediments. The containers were sealed with tight-fitting lids and placed into an incubator (ST2Basic, Pol-Eko Aparatura) set at a daily temperature cycle varying between 26 and 30°C to simulate climate conditions of the northern Red Sea in summer (see [56]). The experimental containers were illuminated on a daily cycle (14h of light and 10h of darkness) to simulate photic conditions in the summer and to promote algal growth. We did not use additional aeration of the containers, since previous studies showed that the use of polypropylene containers ensured sufficient gas exchange and oxic conditions throughout the experiments [10–12, 20, 24]. The oxygen content of each individual container was checked twice during the course of the experiment (after 3 and 6 weeks, respectively; O_2_-Test for aquaria, color scale, JBL) and values did not drop below 183 μmol/kg. At the same interval, pH was checked and values remained around 8 throughout the experiment (pH-indicator paper, neolab). Every 10–11 days, one third of the artificial seawater was replaced with the same amount of fresh Coral Pro, which was aerated until the day before the water change. No additional food was added but algal growth was observed throughout the course of the experiment. The algae and assorted bacteria provided nutrition. Goldstein and Alve [12] and Weinmann and Goldstein [20] also reported signs of feeding in the algal mats of their propagule experiments.

The experimental design resulted in three treatments (3 substrate types) for each of the two subsamples from both sampling locations, which were sampled twice (May and October). Each treatment had a replicate, resulting in a total of 48 treatments. The treatments were harvested after 6 weeks (July 11 and November 27 2017, respectively) by sieving over a 63-μm stainless steel sieve. The use of a 63-μm sieve ensured that foraminifera retained on the sieve must have grown by at least 10 μm over the course of the experiment, which also reduces the possible bias due to different mesh-passing capabilities in differently shaped forms. The resulting material was preserved in 90% ethanol with 2 grams of rose Bengal per liter ethanol. After 2 weeks, the material was washed with tap water over 63 μm and all foraminifera were picked wet. Polythalamous foraminifera were identified, counted and stored in 70% ethanol (S2 Table and S3 Table). The staining of the material allowed distinguishing those specimens that were likely alive at the end of the experiment. Unstained or empty tests reflected reproduction and/or death of specimens during the experiment [11, 48].

All samples were coded for their respective sampling locations, subsamples, season, and substrate types, as listed in Table 2.

**Table 2:** Coding of experimental samples according to site, sampling time, subsample and substrate.

Sample station, sample time	Sample code (Subsamples)	Substrate	Replicate
Chalikiopoulou 1, May 2017	**16**	**M** (muddy substrate)	**a, b**
		**P** (phytal substrate)	
		**R** (rubble substrate)	
Chalikiopoulou 2, May 2017	**17**	**M** (muddy substrate)	**a, b**
		**P** (phytal substrate)	
		**R** (rubble substrate)	
Chalikiopoulou 1, October 2017	**19**	**M** (muddy substrate)	**a, b**
		**P** (phytal substrate)	
		**R** (rubble substrate)	
Chalikiopoulou 2, October 2017	**20**	**M** (muddy substrate)	**a, b**
		**P** (phytal substrate)	
		**R** (rubble substrate)	
Antinioti 1, May 2017	**59**	**M** (muddy substrate)	**a, b**
		**P** (phytal substrate)	
		**R** (rubble substrate)	
Antinioti 2, May 2017	**60**	**M** (muddy substrate)	**a, b**
		**P** (phytal substrate)	
		**R** (rubble substrate)	
Antinioti 1, October 2017	**61**	**M** (muddy substrate)	**a, b**
		**P** (phytal substrate)	
		**R** (rubble substrate)	
Antinioti 2, October 2017	**62**	**M** (muddy substrate)	**a, b**
		**P** (phytal substrate)	
		**R** (rubble substrate)	

### Data analysis and statistics

All foraminiferal faunal analyses such as diversity calculations of assemblages (species richness (S), Shannon (H), and Berger-Parker dominance indices (max p_i_); see [57]), ternary plots, cluster analysis, and multi-dimensional scaling (nMDS) were performed with PAST 3.13 software [58]. Relationships between assemblage characteristics and sampling or experimental variables (sampling site, sampling season, subsamples, substrate type) were tested using analysis of variance (ANOVA) or analysis of similarity (ANOSIM), both performed with PAST 3.13 software. Unless otherwise stated, total abundances of pooled replicates were used in all analyses (stained & unstained assemblages of replicates a & b) as in [12, 20, 24]. For cluster analyses and MDS plots, abundances were square-root transformed and resemblances were calculated using the Bray-Curtis similarity index. Bray-Curtis similarity was also used for ANOSIM analyses. PAST-plots were transferred to Inkscape (V. 0.92.3, www.inkscape.org) and prepared as figures.

For a better understanding of the nature and influence of foraminiferal dispersal, we categorized the benthic taxa found in our study into “autochthonous”, “sporadic”, and “allochthonous” taxa (S4 Table). Based on a classification used by Weinmann and Goldstein ([24] see Table 3 therein), we categorized species as autochthonous if they were found among the stained (living) in situ assemblages or if they were commonly present (>1%) among the dead in situ assemblages. Species that were only sporadically present (<1%) within the in situ dead assemblages were deemed sporadic, indicating that they might be part of the in situ communities at some time of the year and that their status was uncertain. Species that were absent from the in situ assemblages but grew during the experiments were categorized as allochthonous.

**Table 3:** Results of 1-way ANOSIM analyses of in situ benthic assemblages (combined replicates).

	Sampling site	Sampling Season	Subsample	stained vs. dead
*Chalikiopoulou & Antinioti*				
R	0.08	-0.09	-0.09	**0.92**
p	0.14	0.90	0.76	**<0.001**
*Chalikiopoulou only*				
R		-0.02	-0.23	**1**
p		0.46	1	**0.03**
*Antinioti only*				
R		-0.18	-0.16	**1**
p		0.83	0.72	**0.03**

Numbers in bold highlight statistical significances (p<0.05). Count data were square-root transformed and the Bray-Curtis similarity index was used.

For additional analyses, both in situ and experimental benthic taxa were sorted into ecological and functional groups (S5 Table). We applied the ecological groups defined for the calculation of the Foram-AMBI index. Foram-AMBI was based on Borja et al. [59] and developed by the Fobimo group. It was first described by Alve et al. [8] from Atlantic and Arctic areas and further developed for the Mediterranean Sea by Jorissen et al. [9]. To calculate the index, species are distinguished based on their sensitivity to organic enrichment: Group 1 is deemed “sensitive”, Group 2 is deemed “indifferent” and Groups 3–5 are categorized as third-, second-, and first-order opportunists [9]. The latter generally increase in abundances with increasing organic enrichment, whereas the sensitive species will disappear [9]. We assigned our species data to the five ecological groups and calculated their relative abundances within each sample. We only included species that could be clearly assigned according to the species list presented by Jorissen et al. [9], terming the remaining taxa as “unassigned”.

We grouped the benthic foraminiferal taxa into “functional groups” based on their predominant mode of life (S5 Table). We applied this to evaluate the assemblages for possible effects of the three simulated substrate types muddy, phytal, and rubble. For this, we adjusted the categorization of Langer [60], which includes four types of epiphytic species: Epiphytic a (predominantly sessile), epiphytic b (temporary mobile), epiphytic c (predominantly mobile) and epiphytic d (permanently mobile). We further included the groups epifaunal (predominantly on sediment) and infaunal (predominantly within sediment). We included all species in this analysis and chose one predominant life mode for those species that are known to exhibit more than one.

## Results

### In-situ assemblages

In situ assemblages of Chalikiopoulou and Antinioti lagoons contained both benthic and planktonic species. Planktonics made up between 20 and 69% of the combined living and dead assemblages. In all, 120 species of benthic foraminifera were found within in situ assemblages from Chalikiopoulou and Antinioti lagoon. Of those, 30 species were also recorded as stained (possibly living).

Total species richness in Chalikiopoulou lagoon (samples 16, 17, 59, and 60) was 111, with 110 species found dead and 22 species found stained. Percentages of stained benthic and planktonic individuals were low (with 2–6%). Among stained individuals, *Ammonia tepida* was the most common species (17–47%), followed by *Pseudotriloculina rotunda* (7–21%) and *Haynesina depressula* (7–18%). Other common species included *Ammonia parkinsoniana* (6–25%), planktonic species (7–9%), *Asterigerinata mamilla* (3–9%), *Quinqueloculina seminula* (0–8%), and *Rosalina bradyi* (0–8%). Among dead assemblages, planktonic taxa were dominant (21–45%). Further common species included *Ammonia tepida* (5–10%), *Asterigerinata mamilla* (3–7%), *Ammonia parkinsoniana* (3–8%), *Buccella* sp. 1 (2–4%), *Cibicides advenum* (3–4%), and *Rosalina bradyi* (2–4%). ANOSIM analysis between stained and dead benthic assemblages revealed that neither sampling season (May or October, p = 0.46, Table 3) nor subsamples (16 vs. 17 and 59 vs. 60, p = 1) were statistically significant for differences in assemblage composition.

Total species richness in Antinioti lagoon (samples 19, 20, 61, and 62) was lower with 61 species. Of those, 56 species were documented dead and 20 species were found stained. Percentages of stained taxa varied between 2 and 9%. Within stained assemblages, *Ammonia tepida* was dominant (46–68%), followed by planktonic taxa (1–13%). Other common species included *Haynesina depressula* (0–13%), *Elphidium williamsoni* (0–12%), *Quinqueloculina seminula*, and *Aubignyna planidorso* (both 0–10%). The dead assemblages were highly dominated by specimens of planktonic taxa (63–71%). Other common benthic species were *Ammonia tepida* (6–9%) and *Heterolepa* cf. *H*. *subhaidingeri* (4–5%). No significant differences in assemblages were found between sampling seasons or subsamples (samples within each lagoon, see Table 2) (ANOSIM, p = 0.83 for season and p = 0.72 for subsample; Table 3).

Overall diversity was higher in Chalikiopoulou than Antinioti (S1 Table). Species richness (S) in dead assemblages was 69–71 and in stained assemblages was 8–14 in Chalikiopoulou lagoon. Differences between dead and stained assemblages were significant for species richness (ANOVA, F_1,7_ = 214, p<0.001). In Antinioti, species richness among dead taxa was 38–43, and for stained assemblages it was 5–11 (ANOVA for dead vs. stained assemblages: F_1,7_ = 290.4, p<0.001). Sampling sites differed significantly for species richness (F_1,7_ = 46.1, p<0.001) among the dead assemblages. For the stained assemblages, differences between sampling sites were not significant regarding species richness, but sampling season showed a significant effect (F_1,7_ = 8.8, p = 0.03).

### Experimentally grown assemblages

During the course of the experiments (6 weeks), the water quality was tested regularly (S6 Table). Small increases in salinity within individual containers (~0.5–2 ppt) were recorded and were balanced by adding small amounts of distilled water. The experimental containers remained well oxygenized throughout the experiments (concentrations >183 μmol/kg) and pH remained constant (S6 Table). Green and brown algae grew during experiments and built mats on the sediment surface and on rubble (if present). At the termination of the experiments, foraminiferal shells were visible on the sediment surface.

Between 517 and 4487 individuals grew per replicate with 1157–7482 individuals per combined set of replicates (Table 4). Season (time of sampling) was significant for the number of individuals found (ANOVA F_(1,22)_ = 8.3, p = 0.01), whereas numbers did not differ significantly between sampling sites or substratum treatments.

**Table 4:** Number of benthic (including unidentified juveniles) and planktonic individuals that grew in each treatment and diversity indices (only benthic) for experimental assemblages (combined replicates).

	Individuals (benthic)	Individuals (planktonic)	Richness (S)	Shannon (H)	Berger-Parker (max p_i_)
*Chalikiopoulou lagoon*					
**16M**	4414	68	46	2.17	0.27
**16P**	2838	53	31	1.65	0.39
**16R**	3821	139	44	1.83	0.40
**17M**	7401	81	40	2.13	0.25
**17P**	4658	85	45	1.72	0.51
**17R**	6920	8	31	2.06	0.20
**59M**	3350	19	35	2.12	0.28
**59P**	2008	14	29	1.83	0.46
**59R**	1150	7	24	2.01	0.26
**60M**	3201	26	28	1.94	0.35
**60P**	3857	14	22	2.00	0.25
**60R**	3489	20	30	1.96	0.26
*Antinioti lagoon*					
**19M**	3266	21	20	1.58	0.58
**19P**	2906	42	28	1.84	0.39
**19R**	2359	44	26	2.09	0.20
**20M**	3410	16	27	2.10	0.31
**20P**	4571	24	25	1.34	0.64
**20R**	4538	25	25	1.79	0.41
**61M**	2285	7	22	2.06	0.41
**61P**	2362	6	27	2.46	0.20
**61R**	3463	23	26	2.14	0.35
**62M**	2582	14	22	2.27	0.25
**62P**	3914	27	26	1.98	0.46
**62R**	2010	15	22	2.28	0.12

Of the 88 species of benthic foraminifera that grew within the treatments, the majority were rare. Fewer than 10 individuals were found for each of these 45 rare species and for 12 of those, only one individual was found. The species that grew most abundantly in the treatments were *Ammonia tepida*, *Quinqueloculina seminula*, *Pseudotriloculina rotunda*, *Pseudotriloculina* cf. *P*. *oblonga*, *Textularia bocki*, *Haynesina depressula*, *Rosalina bulloides*, and *Miliammina fusca*.

Species richness (S) varied between 20–46 species within combined replicates. Higher species richness was found in treatments with sediments from Chalikiopoulou lagoon (samples 16, 17, 59 and 60; Table 4). Sampling site and season (time of sampling) were significant for species richness (Table 5) Season had a significant effect on Shannon Index values (Table 5), which were higher in spring. If treatments from both sampling sites were analyzed separately, season was significant for species richness in Chalikiopoulou, while it was significant for Shannon diversity in Antinioti (Table 5). No tested variable was significant for dominance (Berger-Parker index; Table 5). The different substrate types revealed no significant effects on faunal diversity (Table 5). Only one significant correlation was found for Shannon diversity between muddy and phytal substrate in treatments from the Chalikiopoulou lagoon (Table 5).

**Table 5:** Results of 1-way ANOVA analyses of diversity indices and number of experimentally grown individuals (combined replicates).

	Sampling site	Sampling Season	Subsample	Substrate
*Chalikiopoulou & Antinioti*				
Species Richness (S)	**F**_**(1,22)**_ **= 13.4, p = 0.001**	**F**_**(1,22)**_ **= 4.8, p = 0.04**		F_(2,21)_ = 0.1, p = 0.93
Shannon Index (H)	F_(1,22)_ = 0.2, p = 0.68	**F**_**(1,22)**_ **= 6.5, p = 0.02**		F_(2,21)_ = 0.2, p = 0.25
Berger-Parker (max p_i_)	F_(1,22)_ = 1.1, p = 0.32	F_(1,22)_ = 1.5, p = 0.24		F_(2,21)_ = 2.2, p = 0.14
*Chalikiopoulou only*				
Species Richness (S)		**F**_**(1,10)**_ **= 11.6, p = 0.01**	F_(1,10)_ = 1.9, p = 0.67	F_(2,9)_ = 0.5, p = 0.62
Shannon Index (H)		F_(1,10)_ = 0.3, p = 0.60	F_(1,10)_ = 0.1, p = 0.73	**F**_**(2,9)**_ = 5.7, p = 0.03*
Berger-Parker (max p_i_)		F_(1,10)_ = 0.2, p = 0.69	F_(1,10)_ = 0.5, p = 0.52	F_(2,9)_ = 2.6, p = 0.13
*Antinioti only*				
Species Richness (S)		F_(1,10)_ = 0.4, p = 0.52	F_(1,10)_ = 2.3, p = 0.16	F_(2,9)_ = 0.4, p = 0.71
Shannon Index (H)		**F**_**(1,10)**_ **= 8.4, p = 0.02**	F_(1,10)_ = 0.6, p = 0.45	F_(2,9)_ = 2.03, p = 0.19
Berger-Parker (max p_i_)		F_(1,10)_ = 1.4, p = 0.26	F_(1,10)_ = 0.2, p = 0.66	F_(2,9)_ = 0.6, p = 0.56

Numbers in bold highlight statistical significances (p<0.05).

*Tukey’s post hoc test Shannon Index and substrate: M/P: p = 0.02; M/R: p = 0.37; P/R: p = 0.19

**Fig 2:**
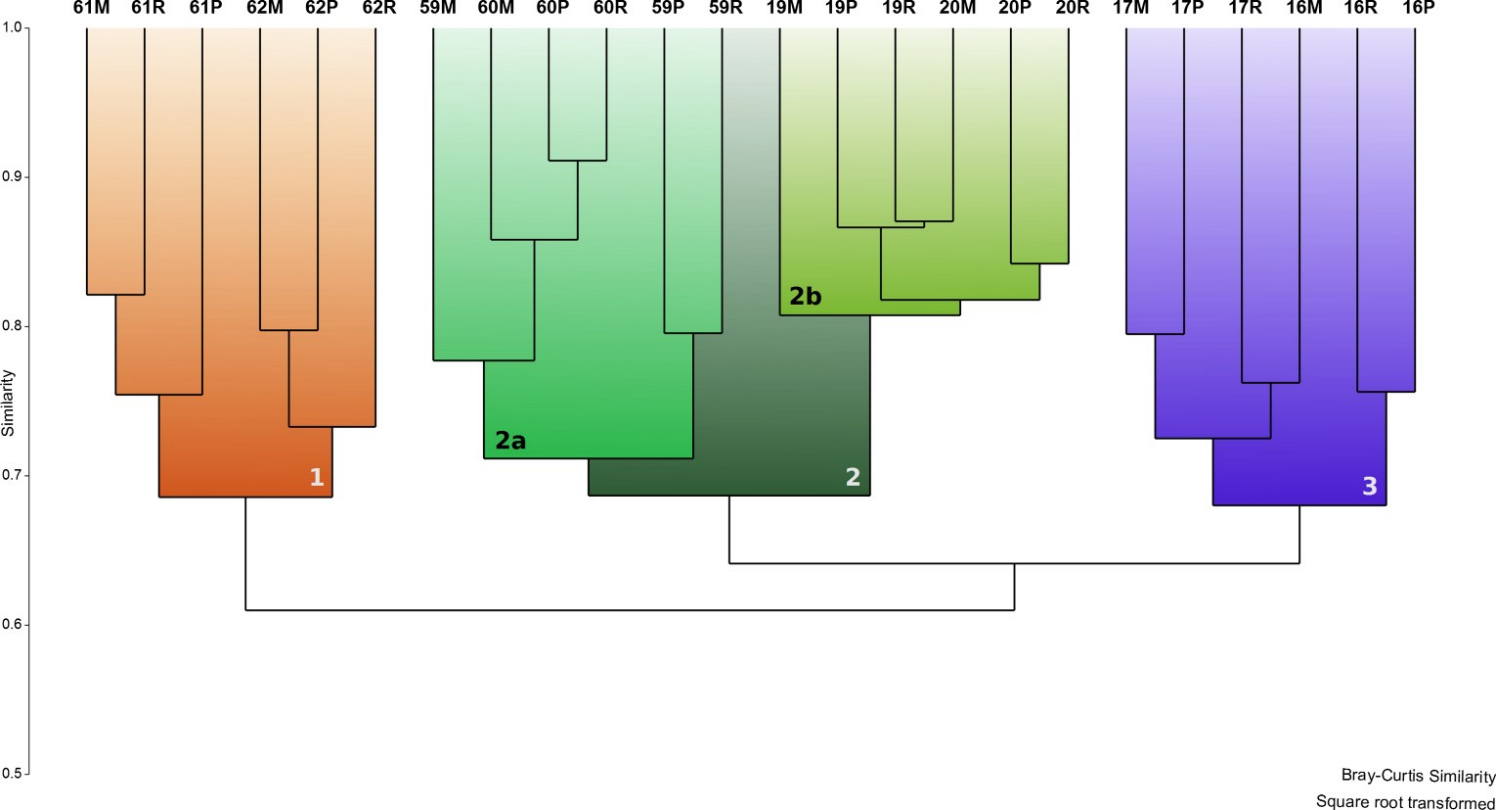
Experimental assemblages (combined replicates) analyzed in a Q-mode cluster. Clusters 1–3 were highlighted at 65% similarity. For sample codes see Table 2.

**Fig 3:**
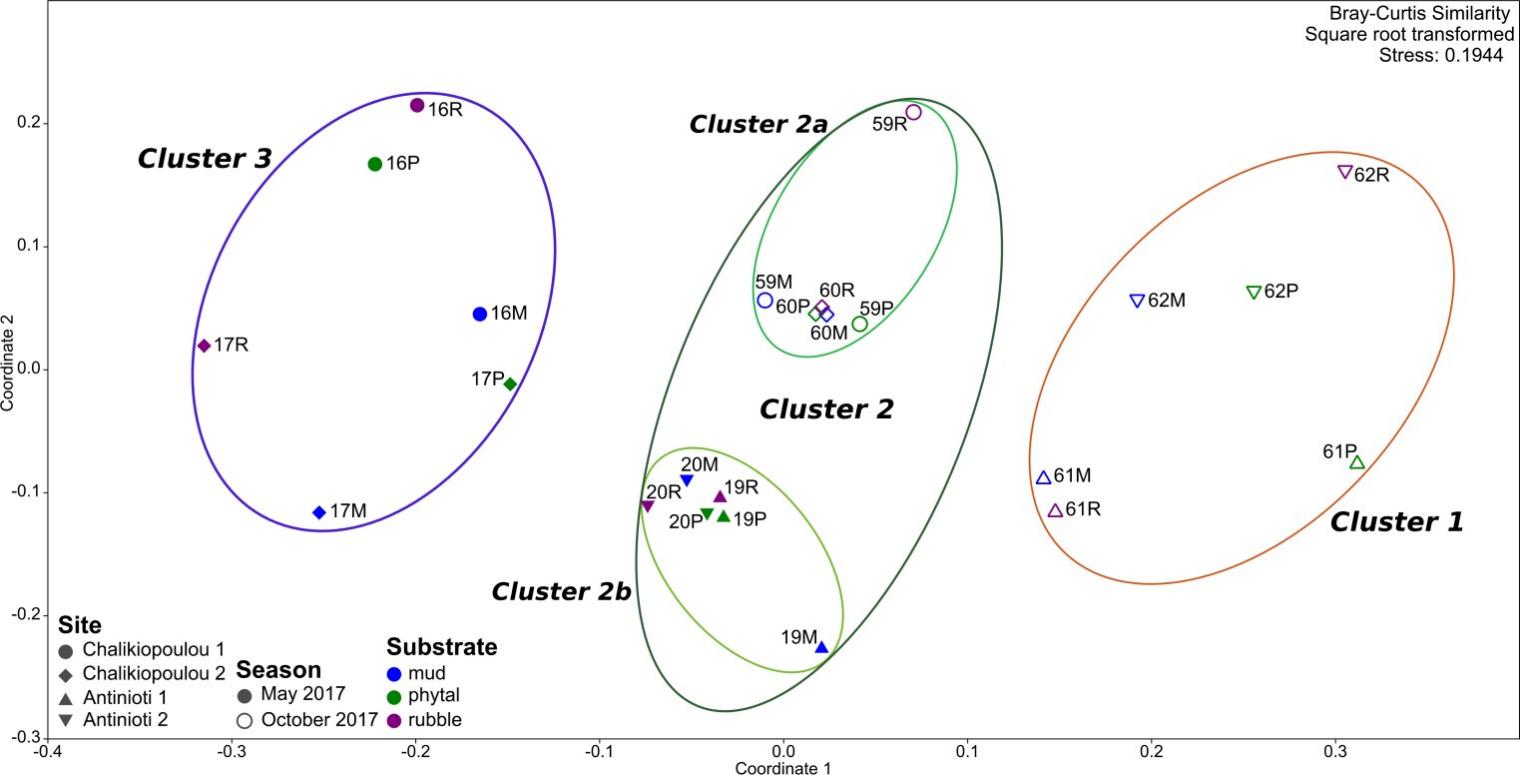
Experimental assemblages (combined replicates) analyzed in an nMDS plot. Superimposed on results are Clusters 1–3 of Fig 2.

Q-mode cluster and nMDS analysis (Bray-Curtis similarity on square-root transformed combined replicates using the 23 most common species) of treatments from both sampling sites revealed three main clusters at 65% similarity (1–3, Figs 2 and 3). Cluster 1 contained all treatments that originated from samples from Antinioti in October (samples 61 and 62) and cluster 3 contained the treatments from Chalikiopoulou samples taken in May. Cluster 2 could be further subdivided into two minor clusters at 70% similarity (2a and 2b, Fig 2), containing results of treatments from the autumn samples from Chalikiopoulou (2a) and the spring samples from Antinioti (2b) respectively. Sampling site and sampling season both had significant effects on assemblage compositions (Table 6). The use of different substrata did not significantly affect the assemblage composition. When analyzed separately, only sampling season was significant for differences in both Chalikiopoulou and Antinioti (Table 6). No significant effects could be observed for subsamples or substrata (Table 6).

**Table 6:** Results of 1-way ANOSIM analyses of experimental assemblages (cumulated replicates).

	Sampling site	Sampling Season	Subsample	Substrate
*Chalikiopoulou & Antinioti*				
R	**0.30**	**0.45**		-0.11
p	**<0.001**	**<0.001**		0.99
*Chalikiopoulou only*				
R		**0.74**	0.14	-0.19
p		**0.003**	0.11	0.94
*Antinioti only*				
R		**0.86**	0.04	-0.2
p		**0.003**	0.26	0.96

Numbers in bold highlight statistical significances (p<0.05). Data have been square root transformed and the Bray-Curtis similarity index was used.

### Comparison of the in situ and experimental assemblages

**Fig 4:**
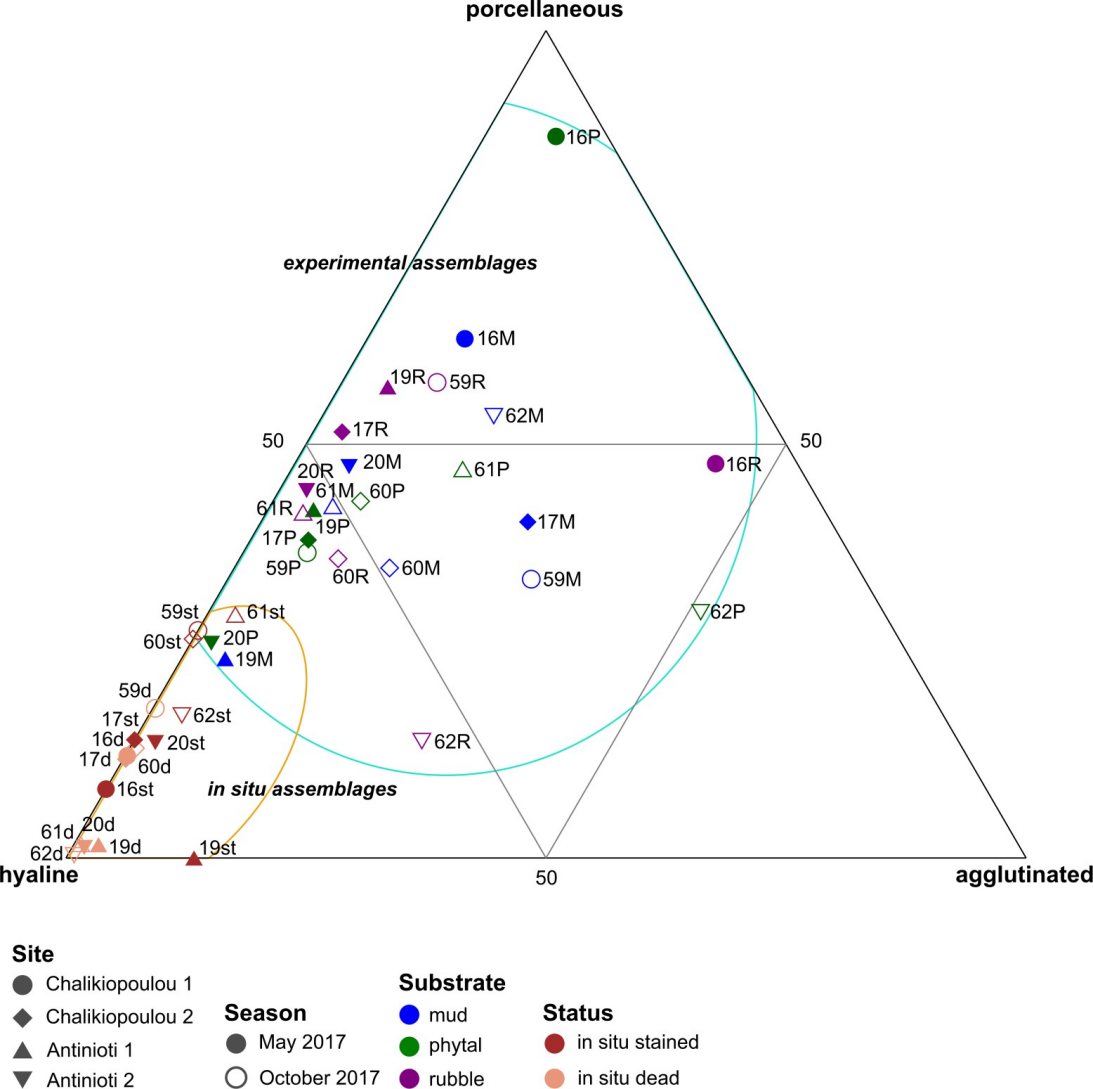
Ternary plot depicting the distribution of foraminiferal shell types within in situ and experimental assemblages (combined replicates). Note that in situ assemblages are distinguished as stained (living) and dead assemblages.

Experimentally grown assemblages differed from in situ assemblages (Fig 4). While both stained and dead in situ assemblages were dominated by hyaline taxa, many experimental assemblages contained more porcellaneous and agglutinated taxa. ANOSIM analysis of the wall structure distributions showed significant differences between in situ dead and experimental assemblages (R = 0.74, p<0.001) as well as in situ stained and experimental assemblages (R = 0.48, p<0.001). In situ stained and dead assemblages were also different from each other (R = 0.24, p = 0.024), but with a lower significance.

Of the 111 benthic species that were found in the in situ samples from Chalikiopoulou lagoon, 63 species also grew in experimental treatments. Twenty of the 22 stained species encountered from the in situ samples also grew during experiments; only *Brizalina* ? sp. 1 and *Conorbella patelliformis* were not found in the experimental assemblages. On the other hand, 19 species grew within experimental treatments from Chalikiopoulou lagoon that were completely absent from the in situ assemblages. Of those, 6 taxa contributed with more than 1% to experimental assemblages within at least one treatment: *Adelosina carinatastriata*, *Ammobaculites* sp. 1, *Miliammina fusca*, *Quinqueloculina limbata*, *Reophax* sp. 1, and *Textularia porrecta*.

In samples from Antinioti lagoon, 61 species were found within the in situ assemblage and 43 of those species also grew in the experimental treatments from that site. Of the 20 stained species from the in situ samples, 17 species also grew during the experiments and only *Brizalina* ? sp. 1, *Bulimina costata* and *Pseudotriloculina jugosa* did not. Within experimental assemblages, 25 species were documented that did not occur within any of the in situ assemblages from Antinioti. Seven of those species contributed more than 1% in a least one treatment: *Ammobaculites* sp. 1, *Bolivina pseudoplicata*, *Cymbaloporetta plana*, *Pseudotriloculina laevigata*, *Quinqueloculina* cf. *Q*. *laevigata*, *Textularia bocki*, and *Triloculina schreiberiana*.

Of the 8 species that grew most abundantly in the experimental treatments, *Ammonia tepida*, *Haynesina depressula*, *Pseudotriloculina* cf. *P*. *oblonga*, *Pseudotriloculina rotunda*, and *Quinqueloculina seminula* were also present within stained in situ assemblages from both sites. *Miliammina fusca* was present in both stained and dead in situ assemblages from Antinioti, but completely absent from all in situ assemblages of Chalikiopoulou lagoon. *Textularia bocki* and *Rosalina bulloides* were absent from the stained assemblages of both sites and *T*. *bocki* was also absent from the dead assemblage of Antinioti.

Within in situ and grown assemblages from Chalikiopoulou, 46 species were defined as autochthonous, 65 as sporadic, and 19 as allochthonous (S4 Table). In Antinioti, we found 37 autochthonous, 24 sporadic, and 25 allochthonous species (S4 Table). Autochthonous species were further divided into those that were present in situ and either did or did not grow during experiments (group 1a and 1b). Sporadic species were also divided into 2 subcategories depending if they did or did not grow during experiments (group 2a and 2b). Allochthonous species represented taxa that were only present within experimental assemblages but did not occur within in situ assemblages from the respective sampling sites (group 3).

**Fig 5:**
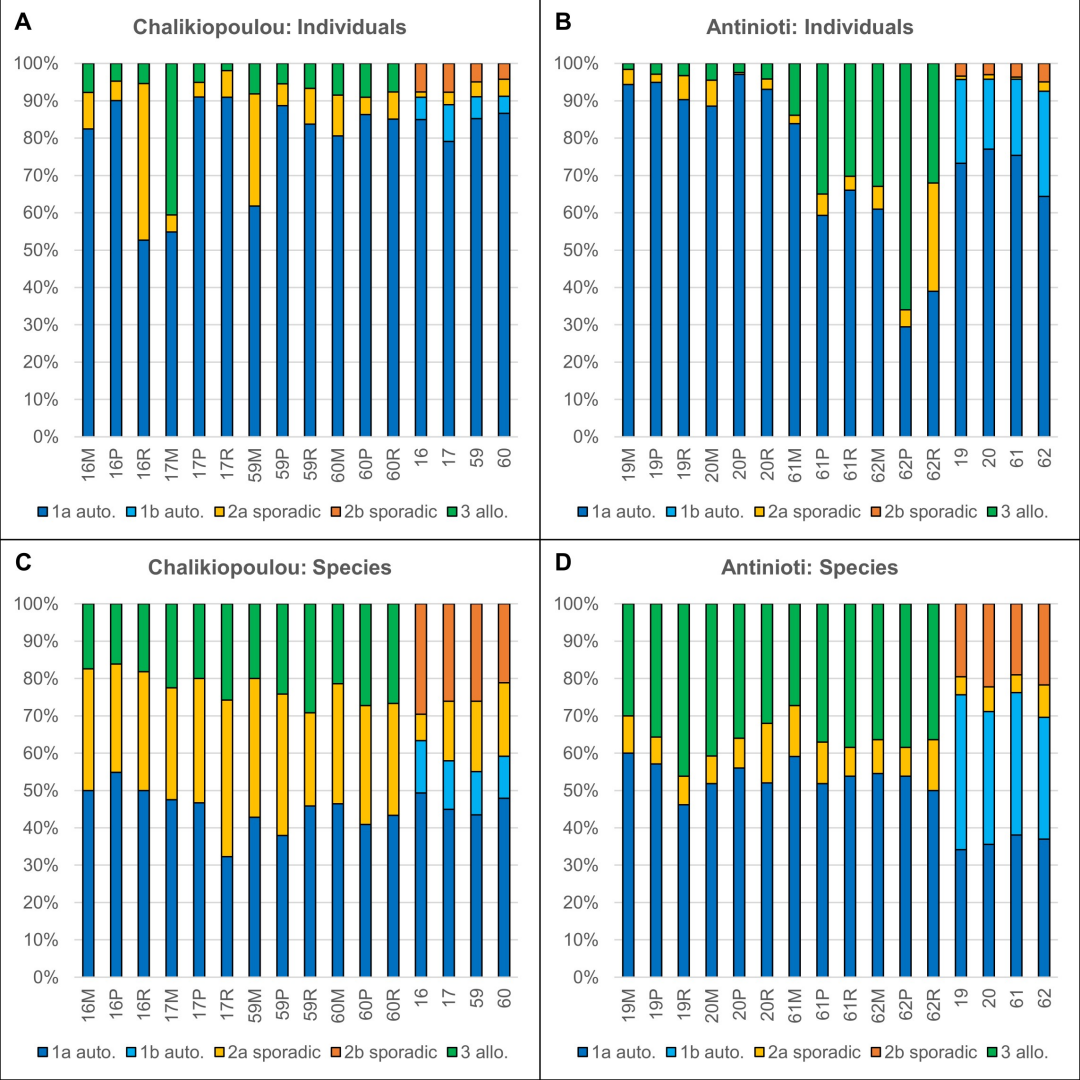
Distribution of relative abundances of autochthonous, sporadic, and allochthonous foraminifera within in situ and experimental assemblages. Group 1a refers to autochthonous taxa that were alive and/or common within in situ assemblages and grew during experiments. Group 1b refers to autochthonous taxa that did not grow during experiments. Group 2a refers to taxa that were sporadically present (<1%) within in situ assemblages and grew during experiments. Group 2b refers to sporadic taxa that did not grow during experiments. Group 3 refers to allochthonous taxa that were absent from in situ assemblages but did grow during experiments. (A) Individuals from Chalikiopoulou lagoon. (B) Individuals from Antinioti lagoon. (C) Species from Chalikiopoulou lagoon. (D) Species from Chalikiopoulou lagoon.

Within in situ assemblages from Chalikiopoulou, 89–91% of all individuals were autochthonous (Fig 5A), although the relative abundances of autochthonous species varied between 55–63% (Fig 5C). In Antinioti, 93–96% of individuals and 70–76% of species were autochthonous (Fig 5B and 5D). The number of autochthonous species that did not grow during experiments (group 1b) was higher at Antinioti than Chalikiopoulou (Fig 5C and 5D). Differences between both sites were significant (ANOSIM R = 0.95, p = 0.03 for individuals and R = 1, p = 0.03 for species). Among the sporadic taxa, the relative abundances of group 2b that did not grow during experiments were higher than sporadic species that did grow (group 2a, Fig 5).

Within experimental assemblages, the abundances of autochthonous individuals were still relatively high, although more variable with 53–91% in Chalikiopoulou (Fig 5A) and 30–97% in Antinioti (Fig 5B). The percentages of species belonging to group 1a (autochthonous species that grew from experiments) were comparable to in situ assemblages (Fig 5C and 5D). Relative abundances of both individuals and species belonging to sporadic species that grew (group 2a) were significantly higher in experimental assemblages (Fig 5). Furthermore, individuals belonging to those species that grew during experiments but were absent from in situ assemblages (allochthonous, group 3) were detected within all treatments from both sites (Fig 5A and 5B). In Antinioti, those individuals were especially abundant in October with 14–66% (Fig 5B). Season was statistically significant for assemblages from Antinioti (R = 0.7, p = 0.002).

**Fig 6:**
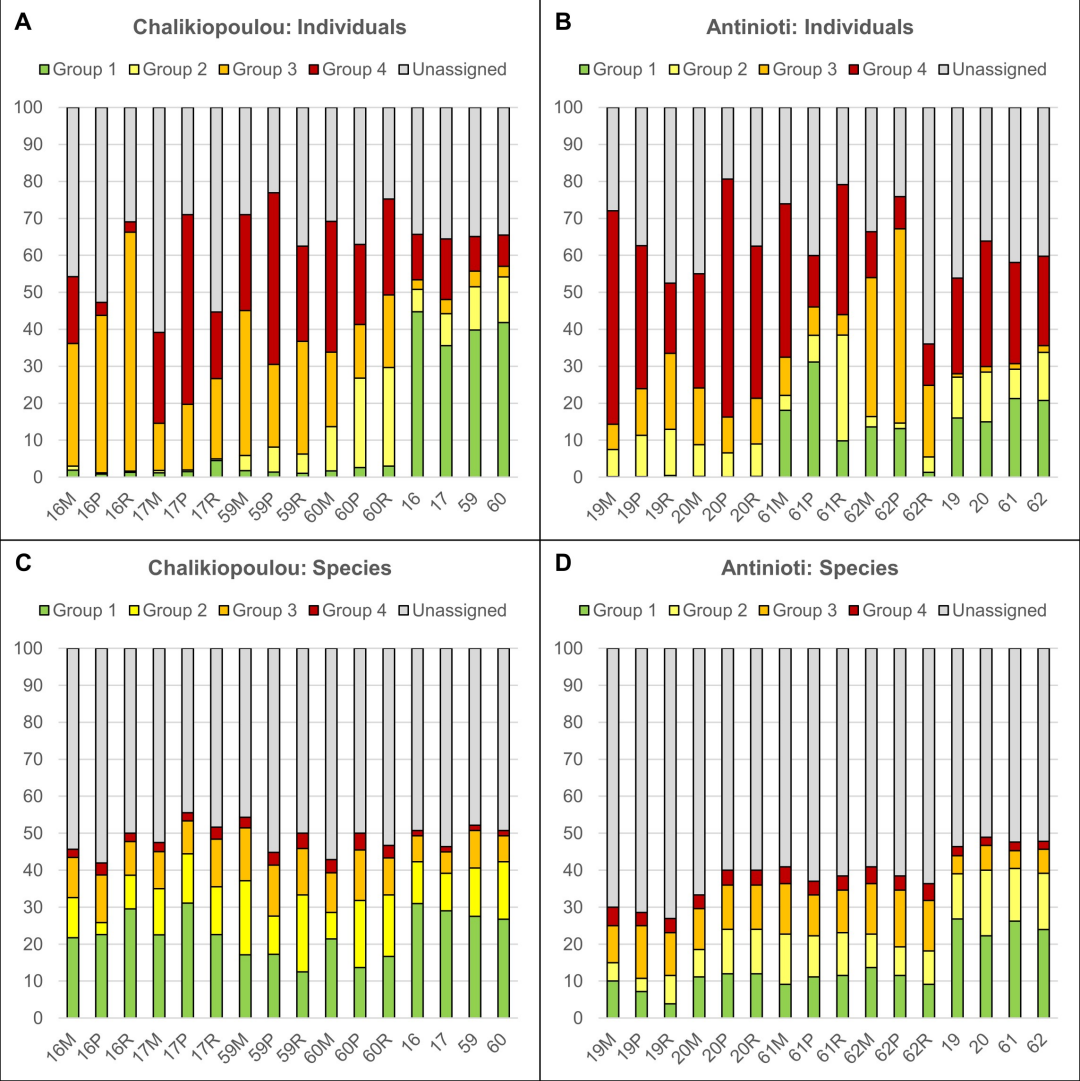
Distribution of relative abundances of foraminifera belonging to four ecological groups based on Foram-AMBI within in situ and experimental assemblages. See text for details on group assignments. (A) Individuals from Chalikiopoulou lagoon. (B) Individuals from Antinioti lagoon. (C) Species from Chalikiopoulou lagoon. (D) Species from Chalikiopoulou lagoon.

For the analysis of ecological groups based on the Foram-AMBI approach, we only grouped species that were mentioned by name in the list of Jorissen et al. [9], leading to relatively high percentages and to a high variability of unassigned taxa (Fig 6, S5 Table). We found 31 species belonging to Group 1, 17 species belonging to Group 2, 9 species belonging to Group 3 and only 1 species (*Ammonia tepida*) belonging to Group 4. We did not find species from Group 5. We plotted the percentages of Foram-AMBI groups among individuals (Fig 6A and 6B) and species (Fig 6C and 6D).

The in situ assemblages of Chalikiopoulou and Antinioti lagoon contained relatively high percentages of individuals belonging to Foram-AMBI Group 1 with 36–45% in Chalikiopoulou and 15–21% in Antinioti (Fig 6A and 6B). Percentages of Groups 2 and 3 were comparable between sites, while Antinioti assemblages contained more Group 4 specimens (*Ammonia tepida*) with 24–34% compared to 8–16% in Chalikiopoulou (Fig 6A and 6B). Differences between sampling sites were statistically significant with regard to specimens (ANOSIM, R = 1, p = 0.03). However, when the distribution of in situ species was analyzed, the sites were not statistically different (p = 0.06, Fig 6C and 6D). Between 22 and 31% of all species were assigned to Group 1, while only 1–2% of all species belonged to Group 4 (only *Ammonia tepida*, Fig 6C and 6D).

The distributions of the four Foram-AMBI Groups within the experimentally grown individuals did not differ significantly between Chalikiopoulou and Antinioti (p = 0.14; Fig 6A and 6B). Overall, high percentages of Groups 3 and 4 grew within the experimental treatments. However, differences between in situ and experimental assemblages were only significant for Chalikiopoulou (R = 0.84, p<0.001; Fig 6A). When examined separately, individuals of both Chalikiopoulou and Antinioti displayed significant differences between sampling seasons (R = 0.38, p = 0.01 for Chalikiopoulou, R = 0.3, p = 0.03 for Antinioti). In Chalikiopoulou samples, relatively few individuals from Groups 1 and 2 were found in assemblages grown in May (0.3–4%). However, Group 2 percentages increased considerably in the experimental assemblages grown in October (Fig 6A). In Antinioti samples, Group 1 was only rare in May experimental treatments (0.1–0.5%) but increased strongly in October (Fig 6B). Subsamples and substrates did not differ significantly. With regard to species distribution (Fig 6C and 6D), only Chalikiopoulou showed a significant difference with regard to sampling season (R = 0.39, p = 0.02). Percentages of Group 1 species were higher in May, while percentages of Groups 3 and 4 species increased in October (Fig 6C).

We further separated the assemblages into six “functional groups” according to their known mode of life (S5 Table). We found species within each of the functional groups: 24 species were epifaunal (predominantly on sediment), 34 species were infaunal (predominantly in sediment), one species belonged to epiphytic a (predominantly sessile), 16 species to epiphytic b (temporary mobile), 12 to epiphytic c (predominantly mobile) and 52 species to epiphytic d (permanently mobile).

**Fig 7:**
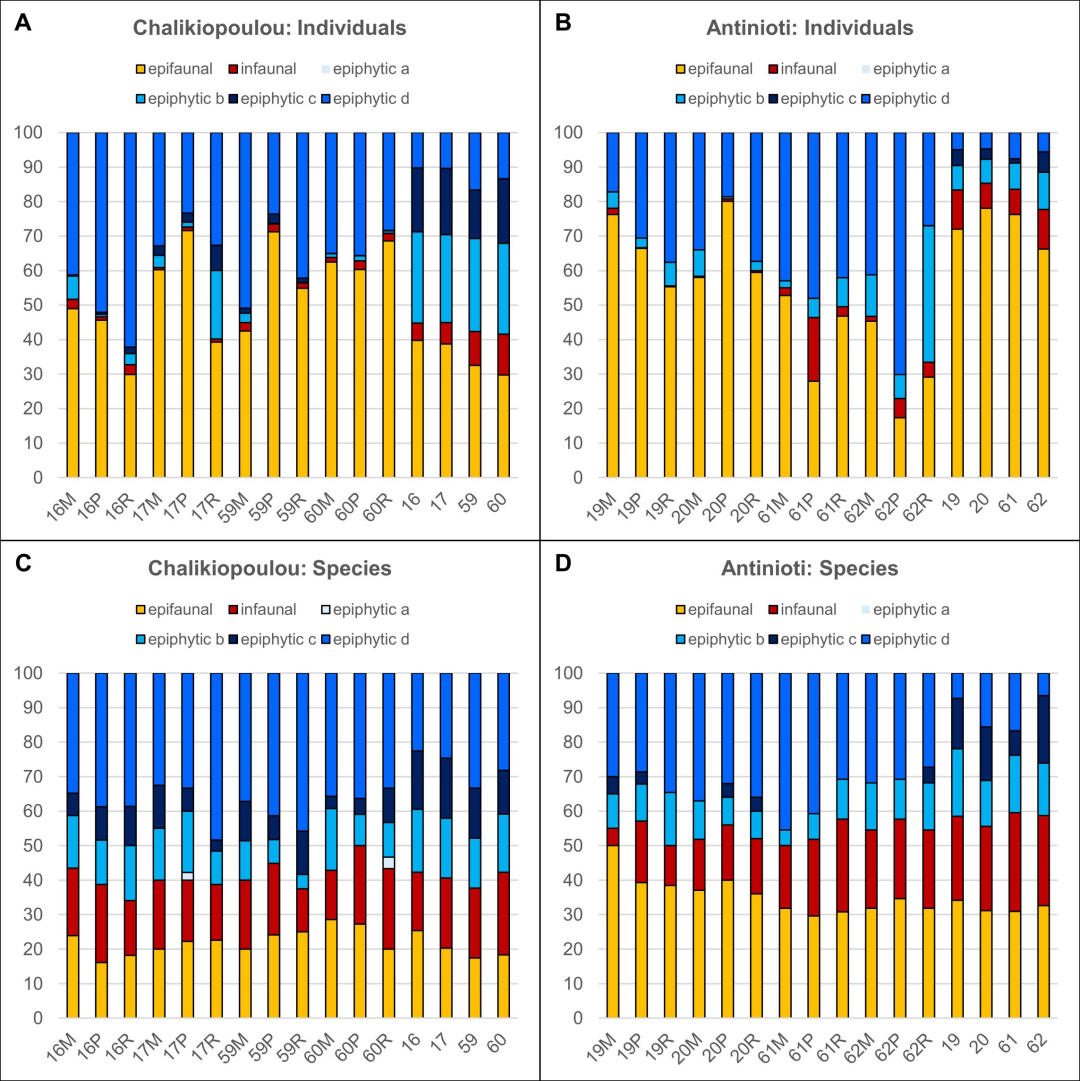
Distribution of relative abundances foraminifera belonging to six functional groups within in situ and experimental assemblages. See text for details on group assignments. (A) Individuals from Chalikiopoulou lagoon. (B) Individuals from Antinioti lagoon. (C) Species from Chalikiopoulou lagoon. (D) Species from Chalikiopoulou lagoon.

The in situ assemblages included specimens from all groups except for epiphytic a. Most were epifaunal with 30–40% in sediments from Chalikiopoulou and 66–78% in those from Antinioti (Fig 7A and 7B). Percentages of infaunal specimens were comparable between the two sites, whereas all epiphytic groups were more common at Chalikiopoulou (Fig 7A and 7B). In situ assemblages differed significantly between sampling sites (ANOSIM, R = 1, p = 0.03) and between relative abundances of species belonging to the functional groups (R = 0.97, p = 0.03), with Antinioti showing overall higher abundances in epi- and infaunal species, but lower abundances in epiphytic c and d (Fig 7C and 7D).

The experimental assemblages were characterized by significantly higher percentages of epiphytic d specimens in addition to overall lower percentages of epiphytic b and c as well as infaunal specimens compared to in situ assemblages (Fig 7A and 7B). Differences between in situ and experimental assemblages were statistically significant for both sites (R = 0.95, p<0.001 for Chalikiopoulou, R = 0.43, p = 0.009 for Antinioti). However, there were no significant differences between sampling sites; epifaunal specimens were common to dominant in both sites. Experimental assemblages from Chalikiopoulou did not show significant differences between season and substrate type. However, subsamples 17 and 59 showed higher percentages of epiphytic c species than subsamples 16 and 60, which highlighted a difference between local subsamples (R = 0.28, p = 0.04). In Antinioti, experimental assemblages grown in October had lower percentages of epifaunal specimens and more epiphytic d specimens (ANOSIM for season: R = 0.43, p = 0.003). As in Chalikiopoulou, there was no statistically significant difference between substrate types. With the relative abundances of species belonging to the functional groups, there was a significant difference between assemblages grown from Chalikiopoulou and Antinioti (R = 0.63, p<0.001), with more epifaunal species and fewer epiphytic c species in Antinioti (Fig 7C and 7D). For both sites, differences in experimental and in situ compositions were significant (R = 0.39, p = 0.02 for Chalikiopoulou, R = 0.89, p<0.01 in Antinioti), but season was only significant for samples from Antinioti (R = 0.51, p = 0.002)

## Discussion

### In situ assemblages

Foraminiferal assemblages in the Chalikiopoulou and Antinioti lagoons of Corfu Island are generally dominated by hyaline taxa such as *Ammonia* spp., *Haynesina depressula*, *Asterigerinata mamilla* and small porcellaneous miliolids such as *Quinqueloculina seminula* and *Pseudotriloculina* spp. As such they are similar to those found in other shallow-water lagoon or sheltered habitats comparable to the studied sites (e.g., [34, 61–64]). Diversity values of the studied sites are also comparable to shallow-marine areas of the Greek mainland (e.g., [39, 65]), and the assemblages reveal a composition that has previously been described from the inlet areas of other lagoonal sites such as the Gulf of Kalloni (Aegean Sea, [34]) or Amvrakikos Gulf (Ionian Sea, [65]). From the latter, it was suggested that the prevalence of miliolids and epifaunal taxa could be attributed to adjacent seagrass and algal vegetation and local hydrodynamic regimes [65], which might also be a factor in the Corfu lagoons, whose shores are extensively vegetated.

The high abundance of planktonic taxa, especially in the in situ assemblages (21–71%) is surprising. Some shells appeared worn, abraded, and “old looking”, suggesting a fossil source. Deposits of planktonic foraminifera are known from Pliocene formations of the island (e.g., [66]), and eroded individuals could have been transported into the lagoons. However, other planktonic specimens appeared more pristine and included a few stained individuals, suggesting a more recent origin. This is particularly surprising, because neither lagoon entrance opens directly into the Ionian Sea, but rather into the Corfu Channel between the island and the mainlands of Albania and Greece. The width of the channel varies between 2 and 22 km and the water depth does not exceed 100 m (portal.emodnet-bathymetry.eu). However, the north-flowing surface current along the Greek coast is probably sufficient to transport small and/or empty planktonic shells from the open sea into the Corfu Channel and subsequently into the lagoons.

Some transport of empty benthic foraminiferal tests into both study sites also likely occurs. The local assemblages consisted of <10% living (stained) individuals. The numbers of species found alive were comparable at both sites (22 and 20 respectively), while 89 species found in Chalikiopoulou and 41 in Antinioti were only present as empty shells. Some of them were abundant and might represent time-averaged accumulations resulting from seasonal blooms. However, the abundance of sporadic species, with rare occurrences in the dead assemblages, were higher in Chalikiopoulou than Antinioti, highlighting the more restricted nature of the latter. While large storm events are uncommon on the eastern coast of Corfu [46], it is possible that specimens are frequently transported into the lagoons from adjacent bays or the deeper channel. This can occur either in suspension or on dislodged seagrass leaves, especially during winter [13, 67].

### Experimentally grown assemblages

The propagule banks of both lagoons contain a high diversity of autochthonous and allochthonous propagules, as evidenced by the 88 species that grew in experimental treatments. Diversity was higher in assemblages that grew from Chalikiopoulou sediments, suggesting that the more open nature of the lagoon facilitates propagule dispersal. Further, the taxonomic composition of the assemblages grown from Chalikiopoulou and Antinioti sediments also differed significantly. Previous studies have already demonstrated the influence of different study sites on the resulting experimental assemblages (e.g. from coastal areas off Georgia and Florida [20]). However, those study sites differed significantly in terms of temperature and salinity. These variables were much more comparable between Chalikiopoulou and Antinioti lagoons, except for the lower salinity values at Antinioti in October 2017 (25 instead of 39–40). The main difference between the two sites appears to be the greater restriction of Antinioti. An effect of site exposure on species richness has previously been described [12]. Although the composition of experimentally grown assemblages differed significantly between the two sites, within the same sampling site, no significant difference was detected between the two subsamples analyzed. This suggests that specimens in propagule banks might be less patchy and more evenly distributed, which would correspond to their “dynamic” nature and the overall strong indications of propagule dispersal.

Both cluster analysis and nMDS plot revealed a significant effect of seasonality, specifically the time of sampling, indicating that propagule banks may vary throughout the year. They are probably influenced by species-specific reproductive cycles, which can be quite variable among taxa [68, 69]. Those are not necessarily bound to annual cyclicity [40, 41], but reproduction can be triggered by other factors, such as a substantial increase in food availability (e.g., [70]). In another example, *Adelosina carinatastriata* exhibited maximum abundances in samples collected during autumn from the Atlantic French coast, suggesting that a large reproduction event occurred during the warmest times of that particular year [71]. In addition, it is likely that seasonal changes in dispersal vectors, such as currents strengths or weather, have an effect on the local propagule assemblages. Differences in the seasonal colonization of natural and artificial seagrasses have previously been described by Ribes et al. [68].

The simulated substrates did not significantly influence the foraminiferal assemblages that developed in the treatments, based on either abundance, diversity or assemblage composition. In case of the artificial substrates (both phytal and rubble), the foraminifera may have rejected both additional substrates and therefore assemblages did not differ from those grown only in the mud or they did not migrate from the muddy substrate to the artificial leaves or rubble. Selectivity in the colonization of artificial substrates was previously demonstrated in an experiment using deep-sea assemblages, where the studied species revealed a preference for one or more types of artificial substrate over others [72]. Ribes et al. [68] showed that epiphytic foraminifera settled preferentially on certain types of artificial substrates. However, they found no significant differences between artificial and natural plant material [68]. Successful settlement on artificial hardgrounds has also been demonstrated (e.g., [73]), although both studies were conducted over longer time periods [68, 73]. On the other hand, variations in temperature and salinity have been shown to strongly influence assemblages grown from propagule experiments [12, 20, 24]. The absence of a significant effect of different simulated substrates on the assemblages grown in our study could suggest that for early life stages, water variables might be more important for the growth and development of foraminiferal specimens.

Within experimental assemblages from both sites and seasons, specimens of *Rosalina bulloides* and *Cymbaloporetta* spp. were found displaying inflated last chambers (i.e., float chambers). Meroplanktonic stages have previously been described for both genera (e.g., [74, 75]) and they are associated with gametogenesis [75, 76]. When asexually produced megalospheres of *Rosalina* (*Tretomphalus*) *bulloides* reach maturity, they produce float chambers and assume a temporary pelagic life-style before gamete release [76]. This adaptation of the life-cycle has also later been described from other taxa such as *Cymbaloporetta* spp. [75]. Many of the specimens with a float chamber found in the present study were empty, suggesting successful gametogenesis during the course of the experiment. The presence of specimens with float chambers at the termination of the experiment suggests the presence of megalospheric juveniles within the original fine fraction of sediment. It is also possible that the megalospheres originated from microspheric propagules, although it cannot be determined if the duration of the experiment provided enough time for the completion of the life cycle [74]. Alve and Goldstein [10] suggested that propagules can be either micro- or megalospheric, although microspheric individuals were more common.

### Comparison of the in situ and experimental assemblages

Experimental assemblages differed significantly from in situ assemblages at both sites. Differences between assemblage compositions could be assessed by analysis of allochthonous taxa, which were absent from the in situ assemblages but grew during experiments. Between 16 and 46% of the experimentally grown species were deemed allochthonous. As such, they represent taxa that were previously “hidden” from the in situ assemblages and could only have been present in the fine fraction of the sediment as small juveniles or propagules. These species included agglutinated taxa such as *Textularia bocki* (allochthonous only in Antinioti) and *Miliammina fusca* (allochthonous only in Chalikiopoulou), which were among the most commonly grown species during the experiments. *Miliammina fusca* is well known from higher estuarine or marsh environments [61, 77] and has previously been found to grow during propagule experiments from Georgia and Florida [12, 20, 24]. It has also been described from marginal areas of the Kalloni Gulf in eastern Greece [32]. As such, small juveniles or propagules could possibly have been transported from the vegetated marginal areas deeper in the lagoon towards the inlet in Chalikiopoulou. Its presence within in situ samples from the Antinioti lagoon suggests that it can occur within the vegetated areas of the Corfu lagoons and is able to tolerate high salinity conditions (at least up to 40 ppt). *Textularia bocki* has been described as an infralittoral or circalittoral taxon in the central Tyrrhenian Sea [78], indicating that its propagules have been transported from outside the lagoon, comparable to findings reported from the US coast [20, 24]. Other allochthonous taxa include several species of small, epiphytic miliolids, which possibly originate from the extensive *Posidonia* meadows along the coast of Corfu Island, where those species have been found (own observations and [79]).

Many species, which were classified as “sporadically present” in the in situ assemblages at both sites were absent from the experimentally grown assemblages. On the other hand, the latter revealed significant percentages of allochthonous taxa not found in the >63 μm fractions at the study sites. This suggests that many sporadic species of the in situ assemblages could have been transported into the lagoons as adults, for example, in suspension or attached to plant debris [13, 14, 67]. The presence of “exotic” taxa belonging to the allochthonous group 3 (S4 Table) suggests that propagules of different species were transported into the lagoons and grew only under experimental conditions. The more restricted nature of Antinioti lagoon is probably responsible for the lower abundance of sporadic and very rare taxa within the in situ assemblages as well as the overall lower diversity. However, the calmer conditions in the more sheltered environment of Antinioti might be advantageous for the settlement of small exotic propagules, which could be an explanation for the high percentages of allochthonous individuals grown from the October samples. At the same time, the number of individuals >63 μm were almost twice as high as in May, which could suggest that the “fine”assemblages were relatively impoverished in autochthonous taxa, which might in turn have resulted in the relatively higher percentages of allochthonous specimens. This would be another argument for the strong influence of seasonality on the composition of local propagule banks.

The ternary plot further highlighted differences in assemblage compositions between in situ and experimental assemblages, which has also been observed in previous studies from the US coast [20, 24]. In those studies, differences in salinities during experiments were interpreted to have been one of the main reasons for assemblage differences between treatments [20, 24]. In our study, the known environmental differences between in situ and experimental conditions were slightly higher temperatures during experiments and differences in substrate. While the three variations in substrate type (muddy, phytal, rubble) did not result in significant differences among assemblages, the restriction towards the size fraction <53 μm could have influenced assemblages grown during experiments. The organic content within sediment is often higher within the fine fractions such as mud or silt, since organic matter is often adsorbed on or within clay minerals (e.g., [5, 9]). As such, using this fine fraction in our experiments could have increased organic enrichment within the experimental treatments.

Our analysis of the distribution of ecological groups based on Foram AMBI within all assemblages showed that most of the experimental assemblages contained higher percentages of groups 3 and 4, which generally react positively to higher organic content [9]. This was mostly observed on the individual level, as the differences were less distinct on the species level (although significant for Antinioti). While we did not encounter first order opportunists (group 5), the significant increase in enrichment-tolerant specimens in Chalikiopoulou treatments suggests an increase in organic content compared to the original sediment composition. Differences in Antinioti lagoon were less distinct, which might be because the original sediment from that site was already more enriched in organic material than Chalikiopoulou due to its lower energy and more restricted circulation conditions (see sample description). Nevertheless, the percentages of group 3 increased during experimental growth. Interestingly, at both sites the percentages of sensitive and indifferent individuals (groups 1 and 2, S5 Table) were higher in experimental treatments grown in October (e.g., *Adelosina carinatastriata*, which has also been described to occur in fine sediments; [39, 71]). This might reflect seasonal variations in organic content, with a potentially higher accumulation during winter months, perhaps due to more land-derived input, and higher rates of consumption over summer. We also found more living individuals >63 μm within in situ assemblages sampled in October, which mainly include *Ammonia tepida*. The higher abundances could have led to more organic material being consumed and thus provided better conditions for the sensitive or indifferent species.

The increased organic content of the experimental conditions did not lead to a significant decrease in oxygen levels during the experiments, which never dropped below 183 μmol/kg. However, together with the increased temperatures, more organic matter would favor group 3 and 4 taxa, with many of them also exhibiting fast growth and reproduction rates [5]. As such, it appears reasonable to assume that they were able to accumulate much faster during the course of the experiment (6 weeks) compared to taxa which might be sensitive to organic content or have slower growth rates. As an example, *Quinqueloculina seminula*, which was among the most common species that grew in our experiments, has previously been described as an early colonizer due to its temporarily opportunistic behavior in terms of growth and reproduction (e.g., [5, 80]). Together with *Ammonia tepida*, it has been deemed as the primary pioneer in paralic environments [80].

Analysis of the functional groups also revealed higher percentages of permanently mobile specimens belonging to the epifaunal and epiphytic d groups. Taxa belonging to the latter group are often associated with plant rhizomes and the surrounding muddy sediments [81]. They can also reproduce within the sediment and, unlike most other epiphytic groups, they have short life-spans [68], which could explain their success within the experimental conditions that featured mostly fine sedimentary substrates, enriched with organic material. Of the eight most abundant species grown from the experiments, all were grouped within either epifaunal or epiphytic d, except *Rosalina bulloides* (epiphytic b). Most epiphytic species are sensitive to eutrophication, although they are less effected by organic accumulation related to the presence of plant debris in seagrass meadows [5]. Since algae, other plant material, and bacteria were probably the main food sources within the experimental conditions, these might favor the growth and development of epifaunal and epiphytic d groups that exhibited the highest abundances within our experimental assemblages.

## Conclusions

In our study on growth experiments on foraminiferal assemblages originating from fine sediments from two shallow-water lagoons of Corfu Island, we gained new insights into the assemblage composition and dynamics of local propagule banks, specifically:

1. Chalikiopoulou and Antinioti lagoons contain diverse but distinct propagule banks, which are influenced by the availability and viability of allochthonous taxa. Within each site, the distribution of small juveniles and propagules appeared to be more uniform (less patchy) than that of adult assemblages.
2. The time of sampling had a significant effect on grown assemblages, suggesting a seasonal influence on the composition and dynamics of local propagule banks, which is probably related to species-specific reproductive cycles and dispersal mechanisms.
3. Although different simulated substrates did not reveal a significant impact, the fine, organic-enriched sediment fraction used in the experiments appeared to influence the resulting foraminiferal assemblages. Together with differences in temperature and salinity, sediment quality (including organic content) may be a key factor in the differences in assemblage compositions between in situ and experimental assemblages. Future experiments that include different sediment compositions could further elucidate this relationship.

Our results shed new light on the structure of foraminiferal propagule banks, their role in local species pools, and the potential responses of foraminiferal communities to ongoing local and global environmental change.

## Supporting information

S1 Table Specimen counts of stained and dead in situ foraminiferal assemblages from both sites. (DOCX)

S2 Table Specimen counts of experimental foraminiferal assemblages grown from Chalikiopoulou lagoon sediments. (DOCX)

S3 Table Specimen counts of experimental foraminiferal assemblages grown from Antinioti lagoon sediments. (DOCX)

S4 Table List of autochthonous, sporadic and allochthonous species from both sites. (DOCX)

S5 Table List of ecological (Foram-AMBI) and functional groups from both sites. (DOCX)

S6 Table Measurements of salinity, pH and oxygen during experiments. (DOCX)

S7 Table Taxonomic list of foraminiferal species found in this study. (DOCX)

## References

[1] Barmawidjaja DM, Jorissen FJ, Puskaric S, van der Zwaan GJ. Microhabitat selection by benthic foraminifera in the northern Adriatic Sea. J Foraminiferal Res. 1992; 22: 297–317.

[2] Murray JW. Ecology and Applications of Benthic Foraminifera. Cambridge: Cambridge University Press; 2006.

[3] Hallock P, Lidz BH, Cockey-Burkhard EM, Donnelly KB. Foraminifera as bioindicators in coral reef assessment and monitoring: The FORAM Index. Environ Monit Assess. 2003; 81: 221–238. 12620018

[4] Carnahan EA, Hoare AM, Hallock P, Lidz BH, Reich CD. Foraminiferal assemblages in Biscayne Bay, Florida, USA: Responses to urban and agricultural influence in a subtropical estuary. Mar Pollut Bull. 2009; 59: 221–233. 10.1016/j.marpolbul.2009.08.008 19744675

[5] Barras C, Jorissen FJ, Labrune C, Andral B, Boissery P. Live benthic foraminiferal faunas from the French Mediterranean Coast. Towards a new biotic index of environmental quality. Ecol Indic. 2014; 36: 719–743.

[6] Debenay JP, Marchand C, Molnar N, Aschenbroich A, Meziane T. Foraminiferal assemblages as bioindicators to assess potential pollution in mangroves used as a natural biofilter for shrimp farm effluents (New Caledonia). Mar Pollut Bull. 2015; 93: 103–120. 10.1016/j.marpolbul.2015.02.009 25758645

[7] Dimiza MD, Triantaphyllou MV, Koukousioura O, Hallock P, Simboura N, Karageorgis AP et al The Foram Stress Index: A new tool for environmental assessment of soft-bottom environments using benthic foraminifera. A case study from the Saronikos Gulf, Greece, Eastern Mediterranean. Ecol Indic. 2016; 60: 611–621.

[8] Alve E, Korsun S, Schönfeld J, Dijkstra N, Golikova E, Hess S et al Foram-AMBI: A sensitivity index based on benthic foraminiferal faunas from North-East Atlantic and Arctic fjords, continental shelves and slopes. Mar Micropaleontol. 2016; 122: 1–12.

[9] Jorissen F, Nardelli MP, Almogi-Labin A, Barras C, Bergamin L, Bicchi E et al Developing Foram-AMBI for biomonitoring in the Mediterranean. Species assignments to ecological categories. Mar Micropaleontol. 2018; 140: 33–45.

[10] Alve E, Goldstein ST. Propagule transport as a key method of dispersal in benthic foraminifera (Protista). Limnol Oceanogr. 2003; 48: 2163–2170.

[11] Alve E, Goldstein ST. The Propagule Method as an Experimental Tool in Foraminiferal Ecology In: Kitazato H, Bernhard JM, editors. Approaches to Study Living Foraminifera. Tokyo: Springer Japan; 2014 pp. 1–12.

[12] Goldstein ST, Alve E. Experimental assembly of foraminiferal communities from coastal propagule banks. Mar Ecol Prog Ser. 2011; 437: 1–11.

[13] Murray JW, Sturrock S, Weston J. Suspended load transport of foraminiferal tests in a tide- and wave-swept sea. J Foraminiferal Res. 1982; 12: 51–65.

[14] Hart M, Molina GS, Smart CW, Widdicombe C. The Western Channel Observatory: Benthic foraminifera in the plankton following storms. Geosci SW England. 2016; 14: 39–45.

[15] Finger K. Tsunami-generated rafting of foraminifera across the North Pacific Ocean. AI. 2018; 13: 17–30.

[16] Guy-Haim T, Hyams-Kaphzan O, Yeruham E, Almogi-Labin A, Carlton JT. A novel marine bioinvasion vector. Ichthyochory, live passage through fish. Limnol Oceanogr. 2017; 2: 81–90.

[17] Hayward BW, Hollis CJ. Brackish foraminifera in New Zealand: A taxonomic and ecologcial review. Micropaleontol. 1994; 40: 185–222.

[18] Almogi-Labin A, Siman-Tov R, Rosenfeld A, Debard E. Occurrence and distribution of the foraminifer *Ammonia beccarii tepida* (Cushman) in water bodies, Recent and Quaternary, of the Dead Sea Rift, Israel. Mar Micropaleontol. 1995; 26: 153–159.

[19] Alve E, Goldstein ST. Dispersal, survival and delayed growth of benthic foraminiferal propagules. J Sea Res. 2010; 63: 36–51.

[20] Weinmann AE, Goldstein ST. Changing structure of benthic foraminiferal communities: Implications from experimentally grown assemblages from coastal Georgia and Florida, USA. Mar Ecol. 2016; 37: 891–906.

[21] Alve E, Goldstein ST. Resting stage in benthic foraminiferal propagules: A key feature for dispersal? Evidence from two shallow-water species. J Micropalaeontol. 2002; 21: 95–96.

[22] Ross BJ, Hallock P. Dormancy in the foraminifera: A review. J Foraminiferal Res. 2016; 46: 358–368.

[23] Schönfeld J. Monitoring benthic foraminiferal dynamics at Bottsand coastal lagoon (western Baltic Sea). J Micropalaeontol. 2018; 37: 383–393.

[24] Weinmann AE, Goldstein ST. Landward directed dispersal of benthic foraminiferal propagules at two shallow-water sites in the Doboy Sound area (Georgia, USA). J Foraminiferal Res. 2017; 47: 325–336.

[25] Duffield CJ, Edvardsen B, Eikrem W, Alve E. Effects of different potential food sources on upper-bathyal benthic foraminifera: An experiment with propagules. J Foraminiferal Res. 2014; 44: 416–433.

[26] Duffield CJ, Hess S, Norling K, Alve E. The response of *Nonionella iridea* and other benthic foraminifera to “fresh” organic matter enrichment and physical disturbance. Mar Micropaleontol. 2015; 120: 20–30.

[27] Buzas MA, Culver SJ. Species pool and dynamics of marine paleocommunities. Science. 1994; 264: 1439–1441. 10.1126/science.264.5164.1439 17838428

[28] Langer MR, Lipps JH. Assembly and persistence of foraminifera in introduced mangroves on Moorea, French Polynesia. Micropaleontol. 2006; 52: 343–355.

[29] Lejeusne C, Chevaldonné P, Pergent-Martini C, Boudouresque CF, Pérez T. Climate change effects on a miniature ocean: the highly diverse, highly impacted Mediterranean Sea. Trends Ecol Evol. 2010; 25: 250–260. 10.1016/j.tree.2009.10.009 19959253

[30] Ferrarin C, Bajo M, Bellafiore D, Cucco A, De Pascalis F, Ghezzo M et al Toward homogenization of Mediterranean lagoons and their loss of hydrodiversity. Geophys Res Lett. 2014; 41: 5935–5941.

[31] Hayward BW, Grenfell H, Cairns G, Smith A. Environmental controls on benthic foraminiferal and thecamoebian associations in a New Zealand tidal inlet. J Foraminiferal Res. 1996; 26: 150–171.

[32] Favry A, Guelorget O., Debenay JP, Lefèbvre A, Perthuisot JP. Répartition et organisation des foraminifères actuels dans le golfe de Kalloni (Grèce). Oceanologica Acta. 1997; 20: 387–397.

[33] Debenay JP, Beck Eichler B, Duleba W, Bonetti C, Eichler-Coelho P. Water stratification in coastal lagoons: its influence on foraminiferal assemblages in two Brazilian lagoons. Mar Micropaleontol. 1998; 35: 67–89.

[34] Debenay JP, Millet B, Angelidis MO. Relationships between foraminiferal assemblages and hydrodynamics in the Gulf of Kalloni, Greece. J Foraminiferal Res. 2005; 35: 327–343.

[35] Koukousioura O, Triantaphyllou MV, Dimiza MD, Pavlopoulos K, Syrides G, Vouvalidis K. Benthic foraminiferal evidence and paleoenvironmental evolution of Holocene coastal plains in the Aegean Sea (Greece). Quat Int. 2012; 261: 105–117.

[36] Culver SJ, Mallinson DJ, Corbett DR, Leorri E, Rouf AA, Shazili NAM et al Distribution of foraminifera in the Setiu estuary and lagoon, Terengganu, Malaysia. J Foraminiferal Res. 2012; 42: 109–133.

[37] Frontalini F, Margaritelli G, Francescangeli F, Rettori R, Armynot du Châtelet E, Coccioni R. Benthic foraminiferal assemblages and biotopes in a coastal lake: the case study of Lake Varano (Southern Italy). Acta Protozool. 2013; 52: 147–160.

[38] Fajemila OT, Langer MR. Ecosystem indicators: Foraminifera, thecamoebians and diatoms from the Ologe Lagoon, Nigeria. Rev Micropaleontol. 2016; 59: 397–407.

[39] Dimiza MD, Koukousioura O, Triantaphyllou MV, Dermitzakis MD. Live and dead benthic foraminiferal assemblages from coastal environments of the Aegean Sea (Greece). Distribution and diversity. Rev Micropaleontol. 2016; 59: 19–32.

[40] Buzas MA, Hayek L-AC, Reed SA, Jett JA. Foraminiferal densities over five years in the Indian River lagoon, Florida: A model of pulsating patches. J Foraminiferal Res. 2002; 32: 68–93.

[41] Morvan J, Debenay JP, Jorissen F, Redois F, Bénéteau E, Delplancke M et al Patchiness and life cycle of intertidal foraminifera: Implication for environmental and paleoenvironmental interpretation. Mar Micropaleontol. 2006; 61: 131–154.

[42] Armynot du Châtelet E, Frontalini F, Francescangeli F. Significance of replicates: Environmental and paleoenvironmental studies on benthic foraminifera and testate amoebae. Micropaleontol. 2017; 63: 257–274.

[43] Boltovskoy E, Lena H. Seasonal occurrences, standing crop and production in benthic foraminifera of Puerto Desado. Contributions Cushman Foundation Foraminiferal Res. 1969; 20: 87–95.

[44] Debenay JP, Bicchi E, Goubert E, Armynot du Châtelet E. Spatio-temporal distribution of benthic foraminifera in relation to estuarine dynamics (Vie estuary, Vendée, W France). Estuar Coast Shelf Sci. 2006; 67: 181–197.

[45] Triantaphyllou MV, Dimiza MD, Koukousioura O, Hallock P. Observations on the life cycle of the symbiont-bearing foraminifer *Amphistegina lobifera* Larsen, an invasive species in coastal ecosystems of the Aegean Sea (Greece, E. Mediterranean). J Foraminiferal Res. 2012; 42: 143–150.

[46] Fischer P, Finkler C, Röbke BR, Baika K, Hadler H, Willershäuser T et al Impact of Holocene tsunamis detected in lagoonal environments on Corfu (Ionian Islands, Greece). Geomorphological, sedimentary and microfaunal evidence. Quat Int. 2016; 401: 4–16.

[47] Schönfeld J, Alve E, Geslin E, Jorissen F, Korsun S, Spezzaferri S, et al The FOBIMO (FOraminiferal BIo-MOnitoring) initiative—Towards a standardised protocol for soft-bottom benthic foraminiferal monitoring studies. Mar Micropaleontol. 2012; 94–95: 1–13.

[48] Murray JW, Bowser SS. Mortality, protoplasm decay rate and reliability of staining techniques to recognize “living” foraminifera: a review. Journal of Foraminiferal Res. 2000; 30: 66–70.

[49] Cherif OH. Die Miliolacea der West-Küste von Naxos (Griechenland) und ihre Lebensbereiche. PhD Thesis, University of Clausthal (Germany). 1970.

[50] Cimerman F, Langer MR. Mediterranean foraminifera. Ljubljana: Academia Scientarium et Artium Slovenica, Opera 30; 1991.

[51] Hottinger L, Halicz E, Reiss Z. Recent foraminiferida from the Gulf of Aqaba, Red Sea. Ljubljana: Slovenska Akademija Znanosti in Umetnosti, Dela Opera, Classics IV, Historia Naturalis; 1993.

[52] Milker Y, Schmiedl G. A taxonomic guide to modern benthic shelf foraminifera of the western Mediterranean Sea. Palaeontol Electronica. 2012; 15: 1–134.

[53] Meriç E, Avşar N, Bergin F. Benthic foraminifera of Eastern Aegean Sea (Turkey) systematics and autoecology. Istanbul: Turkish Marine Research Foundation, Publication Nr 18; 2004.

[54] Meriç E, Avşar N, Yokeş MB, Dinçer F. Atlas of recent benthic foraminifera from Turkey. Micropaleontol. 2014; 60: 211–398.

[55] Mouanga GH. Impact and range extension of invasive foraminifera in the NW Mediterranean Sea: Implications for diversity and ecosystem functioning. PhD Thesis, University of Bonn (Germany). 2018.

[56] Schmidt C, Morard R, Prazeres M, Barak H, Kucera M. Retention of high thermal tolerance in the invasive foraminifera *Amphistegina lobifera* from the Eastern Mediterranean and the Gulf of Aqaba. Mar Biol. 2016; 163: 228.

[57] Hayek LAC, Buzas MA. On the proper and efficient use of diversity measures with individual field samples. J Foraminiferal Res. 2013; 43: 305–313.

[58] Hammer Ø, Harper DAT, Ryan PD. PAST: Paleontological Statistics Software Package for education and data analysis. Palaeontol Electronica. 2001; 4.

[59] Borja A, Franco J, Pérez V. A marine biotic index to establish the ecological quality of soft-bottom benthos within European estuarine and coastal environments. Mar Pollut Bull. 2000; 40: 1100–1114.

[60] Langer MR. Epiphytic foraminifera. Mar Micropaleontol. 1993; 20: 235–265.

[61] Debenay JP, Guillou JJ, Redois F, Geslin E. Distribution trends of foraminiferal assemblages in paralic environments. A base for using foraminifera as bioindicators In: Martin RE, editor. Environmental Micropaleontology, Volume 15 of Topics in Geobiology. New York: Kluwer Academic/Plenum Publishers; 2000 pp. 39–67.

[62] Aiello G, Barra D, Coppa MG, Valente A, Zeni F. Recent infralittoral Foraminiferida and Ostracoda from Porto Cesareo Lagoon (Ionian Sea, Mediterranean). Boll Soc Paleontol Ital. 2006; 45: 1–14.

[63] Berkeley A, Perry CT, Smithers SG, Horton BP, Taylor KG. A review of the ecological and taphonomic controls on foraminiferal assemblage development in intertidal environments. Earth Sci Rev. 2007; 83: 205–230.

[64] Benito X, Trobajo R, Cearreta A, Ibáñez C. Benthic foraminifera as indicators of habitat in a Mediterranean delta Implications for ecological and palaeoenvironmental studies. Estuar Coast Shelf Sci. 2016; 180: 97–113.

[65] Naeher S, Geraga M, Papatheodorou G, Ferentinos G, Kaberi H, Schubert CJ. Environmental variations in a semi-enclosed embayment (Amvrakikos Gulf, Greece)–Reconstructions based on benthic foraminifera abundance and lipid biomarker pattern. Biogeosci. 2012; 9: 5081–5094.

[66] Weltje GJ, de Boer PL. Astronomically induced paleoclimatic oscillations reflected in Pliocene turbidite deposits on Corfu (Greece): Implications for the interpretation of higher order cyclicity in ancient turbidite systems. Geol. 1993; 21: 307–310.

[67] Davaud E, Septfontaine M. Post-mortem onshore transportation of epiphytic foraminifera: Recent example from the Tunisian coastline. J Sediment Res. 1995; A56: 136–142.

[68] Ribes T, Salvado H, Romero J, del Pilar Garcia M. Foraminiferal colonization on artificial seagrass leaves. J Foraminiferal Res. 2000; 30: 192–201.

[69] Murray JW, Alve E. Major aspects of foraminiferal variability (standing crop and biomass) on a monthly scale in an intertidal zone. J Foraminiferal Res. 2000; 30: 177–191.

[70] Schönfeld J, Numberger L. The benthic foraminiferal response to the 2004 spring bloom in the western Baltic Sea. Mar Micropaleontol. 2007; 65: 78–95.

[71] Bouchet VMP, Debenay JP, Sauriau P-G. First report of *Quinqueloculina carinatastriata* (Wiesner, 1923) (Foraminifera) along the French Atlantic Coast (Marennes-Oleron Bay and Ile De Re). J Foraminiferal Res. 2007; 37: 204–212.

[72] Burkett AM, Rathburn AE, Elena Pérez M, Levin LA, Martin JB. Colonization of over a thousand *Cibicidoides wuellerstorfi* (foraminifera. Schwager, 1866) on artificial substrates in seep and adjacent off-seep locations in dysoxic, deep-sea environments. Deep Sea Res Part 1 Oceanogr Res Pap. 2016; 117: 39–50.

[73] Fujita K. A field colonization experiment on small-scale distributions of algal symbiont-bearing larger foraminifera on reef rubble. J Foraminiferal Res. 2004; 34: 169–179.

[74] Rückert-Hilbig A. Megalospheric gamonts of *Rosalina globularis*, *Cymbaloporetta bulloides* and *Cymbaloporetta miletti* (Foraminifera) with differently constructed swimming-apparatus. Tübinger Mikropaläontologische Mitteilungen. 1983; 1:1–69.

[75] Banner FT, Pereira CPG, Desai D. "Tretomphaloid" float chambers in the Discorbidae and Cymbaloporidae. J Foraminiferal Res. 1985; 15: 159–174.

[76] Myers EH. The present state of our knowledge concerning the life cycle of the foraminifera. PNAS. 1938; 24: 10–17. 10.1073/pnas.24.1.10 16588178 PMC1077015

[77] Langer MR, Fajemila OT, Mannl S. Assemblages of recent intertidal mangrove foraminifera from the Akanda National Park, Gabon: Sea level proxies preserved in faunal assemblages. Neues Jahrb Geol Paläontol Abh. 2016; 281: 327–338.

[78] Frezza V, Carboni MG, Matteucci R. Recent foraminiferal assemblages near Ponza Island (Central Tyrrhenian Sea, Italy). Boll Soc Paleontol Ital. 2005; 44: 155–173.

[79] Langer MR, Mouanga GH. Invasion of amphisteginid foraminifera in the Adriatic Sea. Biol Invasions. 2016; 18: 1335–1349.

[80] Debenay JP, Della Patrona L, Goguenheim H. Colonization of coastal environments by foraminifera: Insight from shrimp ponds in New Caledonia (SW Pacific). J Foraminiferal Res. 2009; 39: 249–266.

[81] Mateu-Vicens G, Box A, Deudero S, Rodriguez B. Comparative analysis of epiphytic foraminifera in sediments colonized by seagrass *Posidonia oceanica* and invasive macroalgae *Caulerpa* spp. J Foraminiferal Res. 2010; 40: 134–147.

