# Supplementary material for: Effects of sampling site, season, and substrate on foraminiferal assemblages grown from propagule banks from lagoon sediments of Corfu Island (Greece, Ionian Sea)
Source: PLoS One. 2019 Jun 28;14(6):e0219015. doi: 10.1371/journal.pone.0219015 (PMC6599131; doi:10.1371/journal.pone.0219015)
Supplement: S1 Table — (DOCX) [file pone.0219015.s001.docx]

|  | **Chalikiopoulou lagoon** | | | | | | | | **Antinioti lagoon** | | | | | | | |
| --- | --- | --- | --- | --- | --- | --- | --- | --- | --- | --- | --- | --- | --- | --- | --- | --- |
|  | **May 2017** | | | | **October 2017** | | | | **May 2017** | | | | **October 2017** | | | |
| **Species** | **16st** | **16d** | **17st** | **17d** | **59st** | **59d** | **60st** | **60d** | **19st** | **19d** | **20st** | **20d** | **61st** | **61d** | **62st** | **62d** |
| *Adelosina carinatastriata* |  |  |  |  |  |  |  |  |  |  |  |  | 2 |  | 1 |  |
| *Adelosina cliarensis* |  | 2 |  |  |  |  |  |  |  |  |  |  |  |  |  |  |
| *Adelosina striata* |  | 2 |  | 2 |  |  | 1 |  |  |  |  |  |  |  |  |  |
| *Ammonia beccarii* |  | 1 |  |  |  |  |  |  |  |  |  |  |  |  |  |  |
| *Ammonia inflata* |  | 1 |  |  |  | 2 |  | 2 |  | 20 |  | 20 |  | 16 |  | 14 |
| *Ammonia parkinsoniana* | 3 | 47 |  | 21 | 3 | 24 | 2 | 23 |  | 21 |  | 25 |  | 34 | 1 | 21 |
| *Ammonia tepida* | 2 | 59 | 7 | 61 | 10 | 30 | 8 | 36 | 9 | 75 | 59 | 77 | 18 | 72 | 15 | 63 |
| *Ammonia* sp. 1 |  | 4 |  |  |  |  |  |  |  |  |  |  |  |  |  |  |
| *Amphistegina lobifera* |  |  |  |  |  |  |  |  |  |  |  | 1 |  | 1 |  |  |
| *Asterigerinata mamilla* | 1 | 26 | 1 | 23 | 4 | 29 | 1 | 50 |  | 2 |  | 12 | 1 | 9 |  | 9 |
| *Astrononion stelligerum* |  | 3 |  | 2 |  | 1 |  |  |  |  |  |  |  |  |  |  |
| *Aubignyna planidorso* |  | 7 |  | 6 |  |  |  |  | 1 | 27 |  | 25 | 1 | 22 | 3 | 15 |
| *Bolivina pseudoplicata* |  |  |  |  |  | 1 |  | 3 |  |  |  |  |  |  |  |  |
| *Bolivina variabilis* |  |  |  |  |  |  |  |  |  |  |  | 1 |  |  |  |  |
| *Brizalina* cf. *B. simpsoni* |  |  |  | 1 |  |  |  |  |  |  |  |  |  |  |  |  |
| *Brizalina spathulata* |  | 4 |  |  | 1 | 1 |  | 2 |  | 2 |  |  |  | 1 |  | 3 |
| *Brizalina striatula* |  |  |  | 1 |  | 3 |  |  |  |  |  |  |  |  | 1 |  |
| *Brizalina* ? sp. 1 |  | 1 |  | 2 |  | 2 | 1 | 3 |  | 3 |  | 2 |  | 1 | 1 | 3 |
| *Buccella* sp. 1 |  | 24 |  | 17 | 1 | 15 |  | 31 | 1 | 24 |  | 29 |  | 22 | 1 | 13 |
| *Bulimina costata* |  | 2 |  | 5 |  |  |  | 2 |  | 2 |  | 1 |  | 1 |  | 2 |
| *Bulimina elongata* |  | 1 |  |  |  |  |  |  |  |  |  |  |  |  |  |  |
| *Bulimina* cf. *B. marginata* |  | 1 |  | 2 |  | 1 |  |  |  |  |  | 1 |  |  |  |  |
| *Cancris auriculus* |  |  |  |  |  |  |  | 1 |  |  |  |  |  |  |  |  |
| *Cibicidella variabilis* |  | 2 |  | 2 |  | 4 |  | 5 |  | 1 |  |  |  |  |  |  |
| *Cibicides advenum* |  | 23 |  | 26 | 2 | 19 |  | 21 |  | 9 |  | 6 |  | 9 |  | 10 |
| *Cibicides refulgens* |  | 13 |  | 6 | 1 | 12 | 1 | 10 |  | 3 |  | 2 |  | 2 |  | 2 |
| *Conorbella patelliformis* |  | 1 |  |  | 1 | 2 |  | 1 |  |  |  |  |  |  |  |  |
| *Cycloforina contorta* |  | 1 | 1 | 1 |  |  |  |  |  |  |  |  |  |  |  |  |
| *Cymbaloporetta plana* |  | 2 |  | 3 |  | 1 |  | 3 |  |  |  |  |  |  |  |  |
| *Cymbaloporetta squammosa* |  |  |  | 1 |  |  |  | 1 |  |  |  |  |  |  |  |  |
| *Dentalina* ? sp. 1 |  |  |  | 2 |  |  |  | 1 |  |  |  | 1 |  |  |  |  |
| *Dentalinoides* ? sp. 1 |  |  |  |  |  |  | 1 |  |  | 2 |  |  | 1 | 4 |  |  |
| *Disconorbis bulbosus* |  | 3 |  |  |  | 1 |  | 3 |  |  |  |  |  | 2 |  | 3 |
| *Discorbinella bertheloti* |  | 9 |  | 2 |  | 8 |  | 3 |  | 4 |  | 3 |  | 1 |  | 5 |
| *Elphidium aculeatum* |  | 23 |  | 14 |  | 11 |  | 32 |  |  |  | 3 |  |  |  | 1 |
| *Elphidium* cf. *E. advenum* |  | 12 |  | 4 |  | 4 |  | 7 |  |  |  |  |  |  |  | 5 |
| *Elphidium crispum* |  | 3 |  | 8 | 1 | 4 |  | 4 |  | 1 |  |  |  | 1 |  |  |
| *Elphidium depressulum* |  | 2 | 1 | 1 |  | 9 |  | 15 |  | 5 |  | 1 |  | 1 |  | 2 |
| *Elphidium jenseni* |  | 30 |  | 16 | 1 | 13 | 1 | 20 |  | 1 |  | 1 |  |  |  | 3 |
| *Elphidium* cf. *E. jenseni* |  | 2 |  | 2 |  | 4 |  | 3 |  |  |  |  |  |  |  |  |
| *Elphidium macellum* |  | 4 |  | 12 |  | 6 |  | 8 |  |  |  | 2 |  | 2 |  | 2 |
| *Elphidium williamsoni* |  | 1 |  |  |  |  |  | 8 | 2 | 6 | 1 | 6 |  | 3 | 1 | 2 |
| *Elphidium* sp. 1 |  | 3 |  | 3 |  | 5 |  |  |  |  |  |  |  |  |  | 1 |
| *Elphidium* sp. 2 |  | 3 |  | 5 |  |  |  | 6 |  | 6 |  | 2 |  |  |  | 3 |
| *Eponides concameratus* |  |  |  | 1 |  | 1 |  | 1 |  | 13 |  | 5 |  | 13 |  | 16 |
| *Favulina* sp. 1 |  |  |  |  |  |  |  | 1 |  | 3 |  |  |  |  |  | 1 |
| *Floresina* sp. 1 |  |  |  |  |  | 1 |  |  |  |  |  |  |  |  |  |  |
| *Fursenkoina* sp. 1 |  |  |  | 1 |  |  |  |  |  |  |  |  |  |  |  |  |
| *Gyroidinoides lamarckiana* |  | 4 |  | 4 |  | 1 |  | 2 |  | 2 |  | 2 |  | 1 |  | 3 |
| *Haplophragmoides canariensis* |  |  |  |  |  | 1 |  |  |  |  |  |  |  |  |  |  |
| *Haynesina depressula* | 2 | 6 | 1 | 19 | 3 | 12 | 6 | 10 |  | 3 | 11 | 10 | 2 | 2 |  | 4 |
| *Haynesina* sp. 1 |  | 4 |  |  |  |  |  |  |  |  |  |  |  |  |  |  |
| *Heterolepa* cf. *H. subhaidingeri* |  | 8 |  | 5 |  | 18 |  | 21 |  | 33 |  | 36 |  | 45 |  | 39 |
| *Hoeglundina elegans* |  |  |  | 3 |  | 2 |  | 4 |  |  |  |  |  |  |  |  |
| *Laevipeneroplis karreri* |  |  |  |  |  | 1 |  | 1 |  |  |  |  |  |  |  |  |
| *Lenticulina gibba* |  | 1 |  |  |  |  |  |  |  |  |  |  |  |  |  |  |
| *Lenticulina orbicularis* |  |  |  | 1 |  | 1 |  | 1 |  | 7 |  | 2 |  | 1 |  | 2 |
| *Lobatula lobatula* |  | 4 |  |  |  | 7 |  | 11 |  | 3 |  | 3 |  | 3 |  | 1 |
| *Massilina gualtieriana* |  |  |  |  |  |  |  |  |  |  | 1 |  |  |  |  |  |
| *Massilina secans* |  |  |  |  |  |  |  | 1 |  |  |  |  |  |  |  |  |
| *Melonis pompilioides* |  |  |  | 2 |  | 1 |  | 1 |  | 1 |  | 2 |  | 2 |  | 2 |
| *Miliammina fusca* |  |  |  |  |  |  |  |  |  | 1 | 1 | 2 | 1 |  | 1 | 1 |
| *Miliolinella elongata* |  |  |  |  |  | 1 |  |  |  |  |  |  |  |  |  |  |
| *Miliolinella subrotunda* |  |  |  |  |  |  |  | 1 |  |  |  |  |  |  |  |  |
| *Neoconorbina terquemi* |  | 2 |  |  |  |  |  |  |  | 1 |  |  |  | 1 |  |  |
| *Nonionoides grateloupii* |  |  |  | 1 |  | 1 |  | 2 |  |  |  | 1 |  | 3 |  | 2 |
| *Paracibicides* sp. 1 |  | 4 |  | 5 |  |  |  |  |  |  |  |  |  |  |  |  |
| *Parrina bradyi* |  |  |  | 2 |  | 1 |  |  |  |  |  |  |  |  |  |  |
| *Peneroplis pertusus* |  | 23 |  | 11 |  | 21 |  | 19 |  |  |  |  |  |  |  |  |
| *Peneroplis planatus* |  | 4 |  | 1 |  | 2 |  |  |  |  |  |  |  |  |  |  |
| *Planulina ariminensis* |  | 5 |  | 2 |  | 7 |  | 4 |  | 4 |  | 8 |  | 7 |  | 8 |
| *Polymorphina* sp. 2 |  |  |  |  |  |  |  |  |  |  |  |  |  | 1 |  |  |
| *Poroeponides* ? sp. 1 |  | 1 |  | 3 |  |  |  | 1 |  |  |  | 1 |  |  |  | 1 |
| *Porosononion granosum* |  | 2 |  | 6 |  | 1 |  | 2 |  | 1 |  | 2 |  |  |  | 1 |
| *Porosononion* sp. 1 | 1 | 5 |  | 7 |  |  |  |  |  | 1 |  | 1 |  |  |  | 1 |
| *Protoglobobulimina pupoides* |  | 4 |  | 1 |  | 3 |  | 2 |  |  |  |  |  |  |  |  |
| *Pseudoschlumbergerina ovata* |  |  |  | 1 |  |  |  |  |  |  |  |  |  |  |  |  |
| *Pseudotriloculina jugosa* |  |  |  |  |  |  |  |  |  | 1 | 1 |  |  |  |  |  |
| *Pseudotriloculina* cf. *P. oblonga* |  | 5 |  | 2 |  | 5 | 2 | 2 |  |  | 3 |  | 1 |  | 1 |  |
| *Pseudotriloculina rotunda* |  | 3 | 1 | 6 | 9 | 12 | 7 | 7 |  | 2 | 5 | 2 | 3 | 3 |  |  |
| *Pseudotriloculina* sp. 1 |  | 1 |  | 1 |  | 2 |  | 9 |  |  |  |  |  |  |  |  |
| *Pullenia quadriloba* |  |  |  | 1 |  |  |  |  |  |  |  |  |  |  |  |  |
| *Quinqueloculina auberiana* |  |  |  |  |  | 1 |  |  |  |  |  |  |  |  |  |  |
| *Quinquelcoculina berthelotiana* |  | 1 |  |  |  |  |  |  |  |  |  |  |  |  |  |  |
| *Quinqueloculina bosciana* |  |  |  | 1 |  | 1 |  | 1 |  |  |  |  |  |  |  |  |
| *Quinqueloculina* cf. *Q. irregularis* |  |  |  |  |  |  |  | 2 |  |  |  |  |  |  |  |  |
| *Quinqueloculina* cf. *Q. laevigata* |  |  |  |  |  |  |  | 2 |  |  |  |  |  |  |  |  |
| *Quinqueloculina limbata* |  |  |  |  |  |  |  |  |  |  |  |  | 1 |  |  |  |
| *Quinqueloculina parvula* |  | 2 |  |  |  |  |  |  |  |  |  | 1 |  |  |  |  |
| *Quinqueloculina seminula* | 1 | 7 | 1 | 6 | 2 | 8 |  | 7 |  | 2 | 2 | 1 | 3 | 1 | 3 | 1 |
| *Quinqueloculina stelligera* |  |  |  |  |  | 1 |  | 3 |  |  |  |  |  | 1 |  |  |
| *Quinqueloculina vulgaris* |  |  |  | 1 |  | 2 |  | 2 |  |  |  |  |  |  |  |  |
| *Quinqueloculina* sp. 1 |  |  |  |  |  | 1 |  |  |  |  |  |  |  |  |  |  |
| *Quinqueloculina* sp. 4 |  |  |  |  |  | 1 |  |  |  |  |  |  |  |  |  |  |
| *Reussella spinulosa* |  | 1 |  |  |  |  |  |  |  |  |  |  |  |  |  |  |
| *Rosalina bradyi* | 1 | 22 | 1 | 22 |  | 16 | 1 | 25 |  | 1 |  | 4 |  | 1 |  | 6 |
| *Rosalina bulloides* |  | 1 |  |  |  | 7 |  | 2 |  |  |  |  |  | 1 |  | 1 |
| *Rosalina floridensis* |  | 10 |  | 6 |  |  |  | 1 |  |  |  |  |  |  |  |  |
| *Rosalina macropora* | 1 | 14 |  | 6 |  | 9 | 2 | 11 |  | 2 |  | 1 |  |  |  | 2 |
| *Rosalina* ? cf. *R. suezensis* |  |  |  | 1 |  |  |  |  |  |  |  |  |  |  |  |  |
| *Sigmoilinita costata* |  | 1 |  |  |  |  |  | 1 |  |  |  |  |  |  |  |  |
| *Siphonaperta dilatata* |  | 2 |  | 2 |  | 1 |  | 1 |  |  |  |  |  |  |  |  |
| *Siphonina reticulata* |  | 6 |  | 3 |  | 1 |  | 1 |  | 1 |  | 1 |  | 2 |  | 4 |
| *Sorites orbiculus* |  | 2 |  | 1 |  | 1 |  |  |  |  |  |  |  |  |  |  |
| *Sphaerogypsina* ? sp. 1 |  | 2 |  | 2 |  |  |  |  |  | 1 |  |  |  |  |  | 2 |
| *Spiroloculina angulosa* |  |  |  |  |  | 1 |  |  |  |  |  |  |  |  |  |  |
| *Spiroloculina krumbachi* |  |  |  |  |  |  |  | 1 |  |  |  |  |  |  |  |  |
| *Spiroloculina nitida* |  |  |  | 1 |  |  |  |  |  |  |  |  |  |  |  |  |
| *Spiroloculina ornata* |  | 1 |  |  |  | 1 |  |  |  |  |  |  |  |  |  |  |
| *Textularia bocki* |  |  |  |  |  |  |  | 2 |  |  |  |  |  |  |  |  |
| *Triloculina adriatica* |  |  |  | 3 |  | 1 |  | 1 |  |  |  |  |  |  |  |  |
| *Triloculina* cf. *T. fichteliana* |  | 1 |  |  |  |  |  |  |  |  |  |  |  |  |  |  |
| *Triloculina plicata* |  |  |  |  |  | 1 |  |  |  |  |  |  |  |  |  |  |
| *Triloculina schreiberiana* |  | 1 |  |  |  | 1 |  | 1 |  |  |  |  |  |  |  |  |
| *Triloculina tricarinata* |  | 1 |  | 4 |  |  |  |  |  |  |  |  |  |  |  |  |
| *Trochammina inflata* |  | 1 |  | 1 |  |  |  | 1 | 2 | 7 | 1 | 2 |  | 2 |  | 1 |
| *Uvigerina mediterranea* |  | 2 |  | 3 | 1 | 15 | 1 | 14 |  | 6 |  | 7 |  | 1 |  | 13 |
| *Valvulineria* sp. 1 |  |  |  |  |  |  |  | 1 |  |  |  |  |  |  |  |  |
| *Vertebralina striata* |  |  |  | 2 |  | 2 |  | 3 |  |  |  |  |  |  |  |  |
| Planktonic species |  | 125 | 1 | 323 | 4 | 276 |  | 254 | 2 | 540 | 1 | 528 | 5 | 698 | 2 | 713 |
| Sum | 12 | 611 | 15 | 725 | 44 | 663 | 34 | 744 | 17 | 850 | 87 | 843 | 39 | 993 | 31 | 1007 |
| Sum (only benthics) | 12 | 486 | 14 | 402 | 40 | 387 | 34 | 490 | 15 | 310 | 86 | 315 | 34 | 295 | 29 | 294 |
| Species richness S (only benthics) | 8 | 71 | 8 | 69 | 14 | 69 | 13 | 70 | 5 | 41 | 11 | 42 | 11 | 38 | 11 | 43 |
| Shannon H (only benthics) | 1.98 | 3.53 | 1.67 | 3.56 | 2.25 | 3.63 | 2.2 | 3.57 | 1.21 | 2.87 | 1.2 | 2.82 | 1.72 | 2.66 | 1.74 | 3.01 |
| Berger-Parker max p_i_ (only benthics) | 0.25 | 0.12 | 0.5 | 0.15 | 0.25 | 0.08 | 0.24 | 0.10 | 0.6 | 0.24 | 0.69 | 0.24 | 0.53 | 0.24 | 0.52 | 0.21 |
