# Supplementary material for: Effects of sampling site, season, and substrate on foraminiferal assemblages grown from propagule banks from lagoon sediments of Corfu Island (Greece, Ionian Sea)
Source: PLoS One. 2019 Jun 28;14(6):e0219015. doi: 10.1371/journal.pone.0219015 (PMC6599131; doi:10.1371/journal.pone.0219015)
Supplement: S2 Table — (DOCX) [file pone.0219015.s002.docx]

|  | May 2017 | | | | | | | | | | | | October 2017 | | | | | | | | | | | |
| --- | --- | --- | --- | --- | --- | --- | --- | --- | --- | --- | --- | --- | --- | --- | --- | --- | --- | --- | --- | --- | --- | --- | --- | --- |
|  | Chalikiopoulou 1 | | | | | | Chalikiopoulou 2 | | | | | | Chalikiopoulou 1 | | | | | | Chalikiopoulou 2 | | | | | |
| **Species** | 16Ma | 16Mb | 16Pa | 16Pb | 16Ra | 16Rb | 17Ma | 17Mb | 17Pa | 17Pb | 17Ra | 17Rb | 59Ma | 59Mb | 59Pa | 59Pb | 59Ra | 59Rb | 60Ma | 60Mb | 60Pa | 60Pb | 60Ra | 60Rb |
| *Adelosina carinatastriata* |  |  |  |  |  | 1 |  | 1 | 1 |  |  | 2 | 8 | 25 | 5 | 17 | 8 |  | 22 | 26 | 41 | 41 | 40 | 51 |
| *Adelosina striata* |  | 1 |  |  |  |  |  |  |  |  |  |  |  |  |  | 1 |  |  |  | 1 |  |  | 1 | 3 |
| *Ammobaculites* sp. 1 |  |  |  |  |  |  |  |  |  |  |  |  |  | 1 | 23 |  |  |  |  |  | 3 | 14 | 2 | 2 |
| *Ammonia parkinsoniana* | 4 |  |  |  |  | 2 |  |  | 3 |  |  | 1 | 5 |  |  |  | 1 | 2 | 1 |  |  |  |  |  |
| *Ammonia tepida* | 758 | 15 | 90 | 6 | 10 | 92 | 1777 | 23 | 179 | 2156 | 537 | 699 | 810 | 58 | 796 | 130 | 122 | 171 | 685 | 438 | 367 | 464 | 557 | 345 |
| *Asterigerinata mamilla* | 3 |  | 1 |  |  |  |  | 2 |  |  |  |  |  |  |  |  |  |  |  |  |  |  |  |  |
| *Bolivina pseudoplicata* | 20 | 11 | 9 | 1 | 4 | 2 | 3 | 6 | 1 | 11 | 8 | 4 | 6 | 2 | 6 |  | 3 | 2 |  | 5 | 4 | 5 | 5 | 6 |
| *Brizalina spathulata* | 2 | 10 |  | 3 | 1 | 8 | 2 | 12 | 6 | 3 | 9 | 8 | 3 | 3 | 2 | 4 |  |  |  | 6 | 7 | 1 | 4 | 2 |
| *Brizalina striatula* |  | 10 |  | 1 | 5 | 3 |  | 5 | 1 | 9 | 2 | 14 | 11 | 1 | 1 | 2 | 4 | 3 | 10 | 3 | 12 | 4 | 12 | 2 |
| *Buccella* sp. 1 | 2 | 1 | 2 |  | 1 |  |  |  | 1 | 16 |  |  |  |  |  | 1 |  |  |  |  |  |  |  |  |
| *Cibicidella variabilis* |  |  |  |  |  |  |  |  | 2 |  |  |  |  |  |  |  |  |  |  |  |  |  |  |  |
| *Cibicides advenum* |  | 2 |  | 3 |  | 1 |  |  | 2 | 1 |  |  | 3 |  | 2 |  |  |  | 1 |  | 1 |  |  |  |
| *Cibicides refulgens* |  |  |  |  |  | 3 |  |  |  |  |  |  | 1 |  |  |  |  |  |  |  |  |  | 1 |  |
| *Clavulina angularis* | 1 |  |  |  |  |  |  |  |  |  |  |  |  |  |  |  |  |  |  |  |  |  |  |  |
| *Cornuspira foliacea* |  |  |  |  |  | 1 |  |  |  |  |  |  |  |  |  |  |  |  |  | 1 |  |  |  |  |
| *Cymbaloporetta plana* |  |  |  |  |  |  |  |  |  |  |  |  |  |  |  |  |  |  |  | 24 |  |  |  | 4 |
| *Cymbaloporetta squammosa* | 3 |  |  |  |  |  | 1 | 1 | 1 |  |  | 3 | 1 |  |  |  |  |  |  |  |  |  |  |  |
| *Dentalina* ? sp. 1 |  | 1 |  |  |  |  |  | 1 |  |  |  |  |  |  |  |  |  |  |  |  |  |  |  |  |
| *Dentalinoides* ? sp. 1 |  |  |  | 1 |  | 3 |  |  | 1 | 1 |  |  |  |  |  |  |  |  |  |  |  |  |  |  |
| *Discorbinella bertheloti* |  |  |  |  |  | 5 |  |  |  | 1 |  |  |  |  |  | 1 |  |  |  |  |  |  |  |  |
| *Eggerelloides* sp. 1 |  |  |  |  |  |  |  | 1 |  |  |  |  |  |  |  |  |  |  |  |  |  |  |  |  |
| *Elphidium aculeatum* | 6 |  |  |  |  | 1 |  | 1 |  |  |  |  |  |  |  |  |  |  |  |  |  |  |  |  |
| *Elphidium* cf. *E. advenum* | 1 | 4 |  |  |  | 3 |  | 1 | 1 |  |  |  | 1 |  |  |  | 1 |  |  |  | 1 |  | 2 |  |
| *Elphidium crispum* |  |  |  | 1 |  |  | 1 | 1 | 1 |  |  |  |  |  |  |  |  |  |  |  |  |  |  |  |
| *Elphidium depressulum* |  |  |  |  |  |  |  |  |  |  |  |  | 2 |  |  |  |  |  |  |  |  |  | 2 |  |
| *Elphidium jenseni* | 1 | 1 |  | 1 | 1 |  |  | 10 |  |  |  |  | 2 |  | 5 | 1 | 2 |  |  |  |  |  |  |  |
| *Elphidium williamsoni* |  |  |  | 1 |  |  |  |  |  |  |  |  |  |  |  |  |  |  |  |  |  |  |  | 1 |
| *Elphidium* sp. 2 |  |  |  |  |  | 1 |  |  |  |  |  |  |  |  |  |  |  |  |  |  |  |  |  |  |
| *Eponides concameratus* | 1 |  |  | 1 |  | 1 |  |  |  |  |  |  |  |  |  |  |  |  |  |  |  |  |  |  |
| *Floresina* sp. 1 |  | 4 |  | 2 |  | 1 |  | 6 | 1 | 4 | 1 | 10 | 1 | 1 | 1 | 3 |  |  |  |  |  |  |  |  |
| *Haynesina depressula* | 1 | 3 |  |  |  |  |  | 32 | 1 | 2 |  | 1 | 53 | 67 | 72 | 56 | 25 | 26 | 151 | 225 | 596 | 322 | 593 | 311 |
| *Heterolepa* cf. *H. subhaidingeri* | 4 | 2 |  |  |  | 1 |  |  |  |  |  |  |  |  |  |  |  |  | 1 |  |  |  |  |  |
| *Labrospira subglobosa* |  | 1 |  |  |  |  |  |  |  |  |  |  |  |  |  |  |  |  |  |  |  |  | 1 |  |
| *Lobatula lobatula* |  |  |  |  |  |  |  |  | 1 |  |  |  |  |  |  |  |  |  |  |  |  |  |  |  |
| *Miliammina fusca* |  | 1 | 1 |  | 2 | 1 | 63 | 1672 | 125 | 1 | 5 |  | 10 | 1 | 1 |  |  | 1 | 20 | 87 | 59 | 17 | 19 | 30 |
| *Miliolinella elongata* | 1 | 37 | 1 | 4 | 1 | 4 |  | 10 | 2 | 1 | 2 | 1 | 2 | 11 | 1 |  | 1 | 9 | 1 |  |  |  |  | 1 |
| *Miliolinella subrotunda* | 7 | 23 |  | 12 |  | 2 |  | 24 |  | 3 |  | 3 | 2 |  | 2 |  |  |  |  |  |  | 1 |  | 1 |
| *Neoconorbina terquemi* |  |  |  |  |  |  |  |  | 1 |  |  |  |  |  |  |  |  |  | 1 |  |  |  |  |  |
| *Nonionoides grateloupii* | 2 |  |  |  |  |  |  |  | 1 |  |  |  | 1 |  |  |  |  |  |  |  |  |  |  |  |
| *Paracibicides* sp. 1 | 1 |  |  |  |  | 2 |  | 3 |  |  |  |  |  |  |  |  |  |  |  |  |  |  |  |  |
| *Peneroplis pertusus* | 2 | 1 |  | 2 |  | 2 |  |  | 3 |  |  |  | 1 |  |  |  |  |  | 1 |  |  |  |  |  |
| *Planorbulina mediterranensis* |  |  |  |  |  |  |  |  | 2 |  |  |  |  |  |  |  |  |  |  |  |  |  |  | 2 |
| *Planulina ariminensis* |  | 1 |  |  |  |  | 1 |  |  |  |  |  |  |  |  |  |  |  |  |  |  |  |  |  |
| *Polymorphina* sp. 3 |  |  |  |  |  |  | 3 |  |  |  |  |  |  |  |  |  |  |  |  |  |  |  |  |  |
| *Porosononion* sp. 1 |  |  | 15 |  | 1 | 60 | 136 | 48 | 33 | 87 | 137 | 361 | 45 |  | 37 | 13 | 12 |  | 2 |  |  |  | 1 |  |
| *Pseudotriloculina* cf. *P. oblonga* | 184 | 102 | 156 | 510 | 135 | 95 | 254 | 345 | 159 | 305 | 284 | 335 | 145 | 93 | 115 | 40 | 54 | 28 | 37 | 22 | 97 | 21 | 24 | 18 |
| *Pseudotriloculina rotunda* | 207 | 752 | 208 | 392 | 144 | 380 | 277 | 112 | 214 | 202 | 779 | 370 | 166 | 167 | 82 | 65 | 148 | 104 | 288 | 365 | 468 | 506 | 320 | 288 |
| *Pseudotriloculina* sp. 1 |  | 23 |  | 1 |  |  | 1 | 2 |  |  |  |  |  |  |  |  | 1 |  |  |  |  |  |  |  |
| *Quinquelcoculina berthelotiana* |  |  |  |  |  | 1 |  |  |  |  |  |  |  |  |  |  |  |  |  |  |  |  |  |  |
| *Quinqueloculina bicarinata* |  |  |  | 1 |  |  |  |  |  |  |  |  |  |  |  |  | 1 |  |  |  |  |  |  |  |
| *Quinqueloculina bosciana* |  |  |  |  |  |  |  |  | 1 |  |  |  | 3 |  |  |  | 1 |  |  |  | 2 |  | 8 |  |
| *Quinqueloculina contorta* | 1 |  |  |  |  | 1 |  |  |  |  |  |  |  |  |  |  |  |  |  |  |  |  |  |  |
| *Quinqueloculina* cf. *Q. laevigata* | 10 | 5 |  | 2 | 1 | 11 | 6 | 24 | 16 | 19 | 13 | 282 | 11 | 7 | 1 | 1 | 1 |  | 2 |  | 11 | 6 | 3 | 7 |
| *Quinqueloculina limbata* | 126 | 19 |  | 25 | 2 | 8 | 9 | 1123 | 22 | 8 | 2 | 16 | 92 | 26 | 18 | 25 | 38 |  | 19 | 10 | 21 | 27 | 18 | 1 |
| *Quinqueloculina seminula* | 164 | 988 | 442 | 611 | 370 | 513 | 93 | 624 | 443 | 257 | 746 | 600 | 219 | 139 | 164 | 184 | 146 | 109 | 126 | 183 | 256 | 157 | 250 | 218 |
| *Quinqueloculina stelligera* | 1 | 1 |  | 1 |  |  |  |  |  |  |  |  |  | 1 |  |  |  |  |  |  |  |  |  |  |
| *Quinqueloculina viennensis* | 4 |  |  |  |  | 1 |  | 3 |  | 1 | 4 | 16 | 1 |  |  | 1 |  | 1 |  |  |  |  |  |  |
| *Quinqueloculina vulgaris* |  |  |  |  | 1 |  |  |  |  |  | 1 |  |  |  |  |  |  |  | 1 |  |  |  |  |  |
| *Quinqueloculina* sp. 4 |  |  |  |  |  |  |  |  |  |  | 1 |  |  |  |  |  |  |  |  |  |  |  |  |  |
| *Reophax* sp. 1 | 26 | 27 | 6 | 2 | 36 | 40 | 5 |  | 4 | 2 | 1 | 8 | 49 | 2 |  | 2 | 2 | 3 | 7 | 11 | 29 | 17 | 32 | 5 |
| *Rosalina bradyi* | 3 | 4 | 3 | 1 | 1 | 8 |  | 25 | 11 | 1 | 1 | 2 |  |  |  |  |  |  |  |  |  |  |  |  |
| *Rosalina bulloides* | 32 | 230 | 7 | 3 | 21 | 77 | 95 | 133 | 36 | 5 | 32 | 1318 | 85 |  |  |  | 1 |  | 6 | 1 | 4 | 52 |  | 19 |
| *Rosalina floridensis* | 12 |  |  |  |  |  |  | 1 |  |  |  |  |  |  |  |  |  |  |  |  |  |  |  |  |
| *Rosalina macropora* |  |  |  |  |  |  |  |  | 2 |  |  |  |  |  |  |  |  |  |  |  |  |  |  |  |
| *Siphonaperta dilatata* |  | 1 |  |  |  |  |  |  | 2 |  |  |  |  |  |  |  |  |  |  |  |  |  |  |  |
| *Siphonina reticulata* |  | 1 |  |  |  |  |  |  |  |  |  |  |  |  |  |  |  |  |  |  |  |  |  |  |
| *Spiroloculina antillarum* |  |  |  |  |  |  |  |  |  |  |  | 1 |  |  |  |  |  |  |  |  |  |  |  |  |
| *Spiroloculina cymbium* |  |  |  |  |  |  |  |  |  |  | 1 |  |  |  |  |  |  |  |  |  |  |  |  |  |
| *Spiroloculina nitida* |  |  |  |  |  | 2 |  |  |  | 1 |  |  |  |  |  |  |  |  |  |  |  |  |  |  |
| *Stomatorbina concentrica* |  |  |  |  |  |  |  |  | 2 |  |  |  |  |  |  |  |  |  |  |  |  |  |  |  |
| *Textularia bocki* | 207 | 26 | 5 | 102 | 1444 | 40 | 89 | 103 | 3 | 77 | 4 | 93 | 113 | 823 | 61 | 29 | 38 | 47 | 190 | 129 | 86 | 44 | 133 | 69 |
| *Textularia porrecta* | 104 | 21 | 91 | 4 | 82 | 23 | 81 | 7 | 32 | 27 | 7 | 68 | 35 | 22 | 14 | 3 | 16 | 6 | 33 | 34 | 69 | 10 | 49 | 14 |
| *Textularia* ? *truncata* |  |  |  |  |  |  |  | 1 |  | 2 |  |  |  |  |  |  |  |  |  |  |  |  |  |  |
| *Triloculina schreiberiana* | 8 | 16 |  |  | 10 |  | 20 |  | 17 |  | 9 | 14 | 5 |  | 2 | 3 |  |  |  |  |  |  |  |  |
| *Trochammina inflata* | 26 |  |  |  | 1 | 6 | 35 |  | 5 | 3 | 2 | 20 | 2 | 1 |  | 1 |  |  | 3 |  | 1 | 2 | 2 |  |
| *Uvigerina mediterranea* |  |  |  |  | 1 | 1 | 1 |  |  |  |  |  |  |  |  |  |  |  |  |  |  |  |  |  |
| *Vertebralina striata* |  |  |  |  | 7 | 2 | 1 |  | 1 | 1 | 1 |  |  |  |  | 1 |  |  |  |  |  |  |  |  |
| Planktonic species | 37 | 31 | 16 | 37 | 13 | 126 | 12 | 69 | 58 | 27 | 7 | 1 | 19 |  | 3 | 11 | 7 |  | 18 | 8 | 13 | 1 | 12 | 8 |
| Unidentified juveniles | 84 | 50 | 72 | 34 | 60 | 70 | 29 | 55 | 89 | 22 | 44 | 37 | 4 | 1 | 11 | 2 | 7 | 5 | 13 | 9 | 7 | 4 | 4 | 6 |
| Sum | 2056 | 2426 | 1125 | 1766 | 2355 | 1605 | 2995 | 4487 | 1487 | 3256 | 2640 | 4288 | 1917 | 1452 | 1425 | 597 | 640 | 517 | 1639 | 1588 | 2155 | 1716 | 2095 | 1414 |
| Sum (only benthics) | 2019 | 2395 | 1109 | 1729 | 2342 | 1479 | 2983 | 4418 | 1429 | 3229 | 2633 | 4287 | 1898 | 1452 | 1422 | 586 | 633 | 517 | 1621 | 1580 | 2142 | 1715 | 2083 | 1406 |
| Sum (only benthics & non-juvenile) | 1935 | 2345 | 1037 | 1695 | 2282 | 1409 | 2954 | 4363 | 1340 | 3207 | 2589 | 4250 | 1894 | 1451 | 1411 | 584 | 626 | 512 | 1608 | 1571 | 2135 | 1711 | 2079 | 1400 |
