# Supplementary material for: Effects of sampling site, season, and substrate on foraminiferal assemblages grown from propagule banks from lagoon sediments of Corfu Island (Greece, Ionian Sea)
Source: PLoS One. 2019 Jun 28;14(6):e0219015. doi: 10.1371/journal.pone.0219015 (PMC6599131; doi:10.1371/journal.pone.0219015)
Supplement: S3 Table — (DOCX) [file pone.0219015.s003.docx]

|  | May 2017 | | | | | | | | | | | | October 2017 | | | | | | | | | | | |
| --- | --- | --- | --- | --- | --- | --- | --- | --- | --- | --- | --- | --- | --- | --- | --- | --- | --- | --- | --- | --- | --- | --- | --- | --- |
|  | Antinioti 1 | | | | | | Antinioti 2 | | | | | | Antinioti 1 | | | | | | Antinioti 2 | | | | | |
| **Species** | 19Ma | 19Mb | 19Pa | 19Pb | 19Ra | 19Rb | 20Ma | 20Mb | 20Pa | 20Pb | 20Ra | 20Rb | 61Ma | 61Mb | 61Pa | 61Pb | 61Ra | 61Rb | 62Ma | 62Mb | 62Pa | 62Pb | 62Ra | 62Rb |
| *Adelosina carinatastriata* |  |  |  |  |  |  |  | 1 |  |  |  | 1 | 106 | 140 | 151 | 110 | 176 | 103 | 12 | 1 | 4 | 18 | 4 | 11 |
| *Adelosina striata* | 1 | 2 | 1 | 2 |  |  | 1 | 1 | 1 |  |  |  |  |  |  |  |  |  |  |  |  |  |  |  |
| *Ammobaculites* sp. 1 |  |  |  |  |  |  |  |  |  |  |  |  |  |  | 215 |  | 2 |  | 8 |  | 131 | 1 | 29 | 1 |
| *Ammonia parkinsoniana* | 4 |  | 1 | 2 |  |  | 1 | 5 |  | 1 |  |  |  |  |  |  |  | 1 |  |  | 1 | 1 |  |  |
| *Ammonia tepida* | 696 | 1184 | 370 | 750 | 231 | 213 | 297 | 749 | 1831 | 1101 | 1015 | 847 | 443 | 503 | 299 | 28 | 1122 | 95 | 278 | 40 | 119 | 221 | 50 | 174 |
| *Aubignyna planidorso* |  | 1 | 3 | 4 | 2 |  | 1 | 5 | 1 | 6 |  | 1 |  |  |  | 1 |  |  |  | 1 | 1 |  |  | 1 |
| *Bolivina pseudoplicata* |  |  |  |  |  | 1 | 2 | 2 | 3 | 9 | 2 | 15 | 7 | 9 | 8 | 13 | 16 | 13 | 2 | 2 | 10 | 3 | 8 | 18 |
| *Brizalina difformis* |  |  |  |  |  |  |  |  |  |  |  |  |  |  |  |  |  | 1 |  |  |  |  |  |  |
| *Brizalina spathulata* |  |  | 1 | 1 | 3 | 1 |  | 2 | 5 | 1 | 3 |  | 2 |  | 3 |  | 6 |  | 12 | 1 | 3 | 12 | 4 | 8 |
| *Brizalina striatula* |  |  | 1 |  |  |  |  |  |  |  |  |  | 7 | 10 | 7 | 3 | 18 | 9 | 3 | 4 | 8 | 7 | 3 | 5 |
| *Buccella* sp. 1 | 12 | 4 | 12 | 7 | 3 | 2 | 2 | 3 | 5 | 10 | 3 | 8 | 6 | 41 | 7 |  | 10 | 4 | 6 | 12 | 2 | 3 |  |  |
| *Cibicides advenum* |  |  |  |  | 1 |  |  |  |  |  |  |  |  |  |  |  |  |  |  |  |  |  |  |  |
| *Cornuspira foliacea* | 1 | 23 | 8 | 9 | 10 | 3 | 4 | 20 | 4 | 6 | 2 | 3 |  |  |  |  |  |  |  |  |  |  |  |  |
| *Cymbaloporetta plana* |  |  |  |  |  | 1 |  |  |  |  |  |  |  |  | 1 |  | 109 | 45 | 2 | 162 | 76 | 18 | 176 | 37 |
| *Cymbaloporetta squammosa* |  |  |  | 1 |  |  |  |  |  |  |  |  |  |  |  |  | 15 |  |  | 1 |  | 14 | 10 | 2 |
| *Eggerelloides* sp. 1 |  |  |  |  | 1 |  |  |  |  |  |  |  |  |  |  |  |  |  |  |  | 1 |  |  |  |
| *Elphidium aculeatum* |  |  |  |  |  |  |  |  |  |  | 1 |  |  |  |  |  |  |  |  |  |  |  |  |  |
| *Elphidium* cf. *E. advenum* |  |  |  |  |  |  |  |  | 1 |  |  |  |  |  |  |  |  |  |  |  |  |  |  |  |
| *Elphidium jenseni* |  | 1 |  |  |  |  |  |  |  |  |  |  |  |  |  |  |  |  |  |  |  |  |  | 1 |
| *Elphidium williamsoni* | 45 | 14 | 2 |  |  |  | 4 | 2 | 9 | 2 | 1 |  | 2 | 14 | 31 | 152 | 20 | 2 |  | 4 |  | 37 |  |  |
| *Elphidium* sp. 2 |  |  | 1 |  |  |  |  |  |  |  |  |  |  |  |  |  |  |  |  |  |  |  |  |  |
| *Floresina* sp. 1 |  |  |  |  |  | 1 | 1 |  |  |  |  |  |  |  |  |  |  |  |  |  |  |  |  |  |
| *Haplophragmoides canariensis* | 3 | 1 | 5 | 2 | 2 | 11 | 2 |  |  |  |  |  |  |  |  |  |  |  |  |  |  |  |  |  |
| *Haynesina depressula* | 143 | 96 | 180 | 145 | 195 | 96 | 130 | 157 | 167 | 115 | 187 | 191 | 33 | 39 | 132 | 16 | 108 | 168 | 8 | 60 | 33 | 13 | 21 | 36 |
| *Heterolepa* cf. *H. subhaidingeri* |  |  |  | 1 | 1 |  |  |  |  | 1 |  | 1 |  |  |  |  |  |  |  |  |  |  |  |  |
| *Labrospira subglobosa* |  |  |  | 1 |  |  |  |  |  |  |  |  |  |  |  |  |  |  |  |  |  |  |  |  |
| *Lenticulina orbicularis* |  |  |  | 1 |  |  |  |  |  |  |  |  |  |  |  |  |  |  |  |  |  |  |  |  |
| *Massilina gualtieriana* |  |  |  |  |  |  |  | 1 |  |  |  |  | 1 |  |  | 1 | 1 |  |  |  |  |  |  |  |
| *Miliammina fusca* | 77 | 6 | 20 | 34 | 27 | 23 | 80 | 20 | 7 | 8 | 1 | 8 | 4 | 20 | 8 | 84 | 13 | 5 | 120 | 1 | 8 | 32 | 210 | 3 |
| *Miliolinella elongata* |  |  |  |  |  | 1 |  |  |  |  | 1 |  |  | 11 |  |  |  |  |  |  |  |  |  |  |
| *Miliolinella subrotunda* |  |  |  |  |  |  |  |  | 1 |  |  |  |  |  |  | 1 |  |  | 1 |  |  |  |  |  |
| *Pseudotriloculina laevigata* |  |  |  |  |  |  |  |  |  |  |  |  |  |  |  |  | 684 |  |  |  |  |  |  |  |
| *Pseudotriloculina* cf. *P. oblonga* | 200 | 154 | 186 | 429 | 209 | 263 | 298 | 215 | 190 | 228 | 469 | 510 | 76 | 109 | 31 | 63 | 164 | 42 | 100 | 4 | 28 | 104 | 26 | 61 |
| *Pseudotriloculina rotunda* | 80 | 99 | 95 | 127 | 182 | 127 | 229 | 178 | 136 | 190 | 146 | 270 | 154 | 81 | 66 | 122 | 93 | 39 | 215 | 44 | 17 | 217 | 25 | 56 |
| *Pseudotriloculina* sp. 1 |  |  |  | 1 | 2 |  |  |  |  |  |  |  |  |  |  |  |  |  |  |  |  |  |  |  |
| *Pyrgo elongata* |  |  |  |  |  |  |  | 1 |  |  |  |  |  |  |  |  |  |  |  |  |  |  |  |  |
| *Quinqueloculina jugosa* |  |  |  |  |  |  |  | 31 |  |  |  |  |  | 1 |  | 1 |  |  |  |  |  |  |  |  |
| *Quinqueloculina* cf. *Q. laevigata* | 1 |  |  | 1 | 7 | 4 |  |  | 2 |  |  | 6 | 6 | 161 | 33 | 437 | 42 | 18 | 84 | 251 | 21 | 467 | 11 | 1 |
| *Quinqueloculina limbata* | 6 | 3 | 4 | 12 | 44 | 16 | 123 | 20 | 20 | 12 | 31 | 93 | 15 | 0 | 8 | 3 | 1 | 1 | 13 | 7 | 4 | 43 | 6 | 20 |
| *Quinqueloculina parvula* |  |  |  |  |  |  |  |  |  |  |  | 2 |  | 3 |  | 1 |  |  |  |  |  |  |  |  |
| *Quinqueloculina seminula* | 90 | 120 | 131 | 206 | 311 | 147 | 271 | 196 | 217 | 160 | 183 | 219 | 66 | 39 | 41 | 29 | 58 | 17 | 588 | 45 | 33 | 190 | 26 | 35 |
| *Reophax* sp. 1 |  |  |  |  |  |  |  |  | 3 |  |  |  |  |  | 1 |  |  | 8 |  |  | 4 | 1 | 10 | 1 |
| *Rosalina bulloides* | 84 | 46 | 6 | 56 | 32 | 114 | 148 | 86 | 5 | 12 | 116 | 1 | 5 | 42 | 73 | 57 | 31 | 92 | 75 | 69 | 57 | 106 | 370 | 199 |
| *Rosalina floridensis* |  |  |  |  |  |  |  | 1 |  |  |  |  |  |  |  |  |  |  |  |  |  |  |  |  |
| *Spiroloculina angulosa* |  |  |  |  |  |  |  |  |  |  |  |  |  |  |  | 1 |  |  |  |  |  |  |  |  |
| *Textularia bocki* | 7 | 6 | 12 | 11 | 15 | 7 | 38 | 11 | 52 | 7 | 86 | 10 | 6 | 109 | 89 | 12 | 17 | 71 | 258 | 68 | 753 | 1055 | 299 | 21 |
| *Textularia porrecta* | 6 | 1 | 17 | 8 | 5 | 3 | 3 | 29 | 12 | 3 | 2 |  |  | 6 | 10 | 1 | 2 | 1 | 5 | 1 | 3 | 13 | 9 | 10 |
| *Triloculina schreiberiana* |  |  | 4 |  | 3 |  | 3 | 1 | 4 | 4 | 61 |  |  |  |  |  |  |  |  |  |  |  |  |  |
| *Trochammina inflata* | 25 | 16 | 17 | 10 | 9 | 16 | 6 | 10 | 3 | 4 | 5 | 18 |  | 4 | 1 |  | 3 | 10 |  |  | 2 | 2 | 5 | 5 |
| *Valvulineria* sp. 1 |  |  |  |  |  |  |  |  |  |  |  | 1 |  |  |  |  |  |  |  |  |  |  |  |  |
| Planktonic species | 14 | 7 | 16 | 26 | 27 | 17 | 4 | 12 | 12 | 12 | 19 | 6 |  | 7 | 1 | 5 | 16 | 7 | 9 | 5 | 13 | 14 | 8 | 7 |
| Unidentified juveniles | 4 | 4 | 5 | 3 | 1 | 13 | 10 | 9 | 4 | 8 | 11 | 7 | 1 | 3 |  | 11 | 1 | 6 | 14 |  | 2 | 14 | 2 |  |
| Sum | 1499 | 1788 | 1098 | 1850 | 1323 | 1080 | 1658 | 1768 | 2695 | 1900 | 2345 | 2218 | 940 | 1352 | 1216 | 1152 | 2728 | 758 | 1813 | 783 | 1334 | 2607 | 1312 | 713 |
| Sum (only benthics) | 1485 | 1781 | 1082 | 1824 | 1296 | 1063 | 1654 | 1756 | 2683 | 1888 | 2326 | 2212 | 940 | 1345 | 1215 | 1147 | 2712 | 751 | 1804 | 778 | 1321 | 2593 | 1304 | 706 |
| Sum (only benthics & non-juvenile) | 1481 | 1777 | 1077 | 1821 | 1295 | 1050 | 1644 | 1747 | 2679 | 1880 | 2315 | 2205 | 939 | 1342 | 1215 | 1136 | 2711 | 745 | 1790 | 778 | 1319 | 2579 | 1302 | 706 |
