# Supplementary material for: Effects of sampling site, season, and substrate on foraminiferal assemblages grown from propagule banks from lagoon sediments of Corfu Island (Greece, Ionian Sea)
Source: PLoS One. 2019 Jun 28;14(6):e0219015. doi: 10.1371/journal.pone.0219015 (PMC6599131; doi:10.1371/journal.pone.0219015)
Supplement: S4 Table — (DOCX) [file pone.0219015.s004.docx]

|  | **Chalikiopoulou lagoon** | | **Antinioti lagoon** | |
| --- | --- | --- | --- | --- |
|  | **Group**:  autochthonous (1a, b),  sporadic (2a, b),  allochthonous (3) | **Growth month:** May (M)  October (O) | **Group**:  autochthonous (1a, b),  sporadic (2a, b),  allochthonous (3) | **Growth month:** May (M)  October (O) |
| *Adelosina carinatastriata* | 3* | M&O | 1a | M&O |
| *Adelosina cliarensis* | 2b | / |  |  |
| *Adelosina striata* | 2a | M&O | 3 | M |
| *Ammobaculites* sp. 1 | 3 | O | 3* | O |
| *Ammonia beccarii* | 2b | / |  |  |
| *Ammonia inflata* | 2b | / | 1b | / |
| *Ammonia parkinsoniana* | 1a | M&O | 1a | M&O |
| *Ammonia tepida* | 1a | M&O | 1a | M&O |
| *Ammonia* sp. 1 | 2b | / |  |  |
| *Amphistegina lobifera* |  |  | 2b | / |
| *Asterigerinata mamilla* | 1a | M | 1b | / |
| *Astrononion stelligerum* | 2b | / |  |  |
| *Aubignyna planidorso* | 1b | / | 1a | M&O |
| *Bolivina pseudoplicata* | 2a* | M&O | 3* | M&O |
| *Bolivina variabilis* |  |  | 2b | / |
| *Brizalina difformis* |  |  | 3 | O |
| *Brizalina* cf. *B. simpsoni* | 2b | / |  |  |
| *Brizalina spathulata* | 1a | M&O | 2a | M&O |
| *Brizalina striatula* | 2a* | M&O | 1a | M&O |
| *Brizalina* ? sp. 1 | 1b | / | 1b | / |
| *Buccella* sp. 1 | 1a | M&O | 1a | M&O |
| *Bulimina costata* | 1b | M&O | 1b | / |
| *Bulimina elongata* | 2b | / |  |  |
| *Bulimina* cf. *B. marginata* | 2b | / | 2b | / |
| *Cancris auriculus* | 2b | / |  |  |
| *Cibicidella variabilis* | 1a | M | 2b | / |
| *Cibicides advenum* | 1a | M&O | 1a | M |
| *Cibicides refulgens* | 1a | M&O | 1b | / |
| *Clavulina angularis* | 3 | M |  |  |
| *Conorbella patelliformis* | 1b | / |  |  |
| *Cornuspira foliacea* | 3 | M&O | 3* | M |
| *Cycloforina contorta* | 2b | / |  |  |
| *Cymbaloporetta plana* | 1a | O | 3* | M&O |
| *Cymbaloporetta squammosa* | 2a | M&O | 3 | M&O |
| *Dentalina* ? sp. 1 | 2a | M | 2b | / |
| *Dentalinoides* ? sp. 1 | 1a | M | 1b | / |
| *Disconorbis bulbosus* | 2b | / | 2b | / |
| *Discorbinella bertheloti* | 1a | M&O | 1b | / |
| *Eggerelloides* sp. 1 | 3 | M | 3 | M&O |
| *Elphidium aculeatum* | 1a | M | 2a | M |
| *Elphidium* cf. *E. advenum* | 1a | M&O | 1a | M |
| *Elphidium crispum* | 1a | M | 2b | / |
| *Elphidium depressulum* | 1a | O | 1b | / |
| *Elphidium jenseni* | 1a | M&O | 2a | M&O |
| *Elphidium* cf. *E. jenseni* | 1b | / |  |  |
| *Elphidium macellum* | 1b | / | 2b | / |
| *Elphidium williamsoni* | 1a | M&O | 1a | M&O |
| *Elphidium* sp. 1 | 1b | / | 2b | / |
| *Elphidium* sp. 2 | 1a | M | 1a | M |
| *Eponides concameratus* | 2a | M | 1b | / |
| *Favulina* sp. 1 | 2b | / | 2b | / |
| *Floresina* sp. 1 | 2a | M&O | 3 | M |
| *Fursenkoina* sp. 1 | 2b | / |  |  |
| *Gyroidinoides lamarckiana* | 1b | / | 1b | / |
| *Haplophragmoides canariensis* | 2b | / | 3 | M |
| *Haynesina depressula* | 1a | M&O | 1a | / |
| *Haynesina* sp. 1 | 2b | / |  |  |
| *Heterolepa* cf. *H. subhaidingeri* | 1a | M&O | 1a | M |
| *Hoeglundina elegans* | 2b | / |  |  |
| *Labrospira subglobosa* | 3 | M&O | 3 | M |
| *Laevipeneroplis karreri* | 2b | / |  |  |
| *Lenticulina gibba* | 2b | / |  |  |
| *Lenticulina orbicularis* | 2b | / | 1a | M |
| *Lobatula lobatula* | 1a | M | 1b | / |
| *Massilina gualtieriana* |  |  | 1a | M&O |
| *Massilina secans* | 2b | / |  |  |
| *Melonis pompilioides* | 2b | / | 1b | / |
| *Miliammina fusca* | 3* | M&O | 1a | M&O |
| *Miliolinella elongata* | 2a* | M&O | 3 | M&O |
| *Miliolinella subrotunda* | 2a* | M&O | 3 | M&O |
| *Neoconorbina terquemi* | 2a | M&O | 2b | / |
| *Nonionoides grateloupii* | 2a | M&O | 2b | / |
| *Paracibicides* sp. 1 | 1a | M&O |  |  |
| *Parrina bradyi* | 2b | / |  |  |
| *Peneroplis pertusus* | 1a | M&O |  |  |
| *Peneroplis planatus* | 2b | / |  |  |
| *Planorbulina mediterranensis* | 3 | M&O |  |  |
| *Planulina ariminensis* | 1a | M | 1b | / |
| *Polymorphina* sp. 2 |  |  | 2b | / |
| *Polymorphina* sp. 3 | 3 | M |  |  |
| *Poroeponides* ? sp. 1 | 2b | / | 2b | / |
| *Porosononion granosum* | 1b | / | 2b | / |
| *Porosononion* sp. 1 | 1a | M&O | 2b | / |
| *Protoglobobulimina pupoides* | 1b | / |  |  |
| *Pseudoschlumbergerina ovata* | 2b | / |  |  |
| *Pseudotriloculina jugosa* |  |  | 1b | / |
| *Pseudotriloculina laevigata* |  |  | 3* | O |
| *Pseudotriloculina* cf. *P. oblonga* | 1a | M&O | 1a | M&O |
| *Pseudotriloculina rotunda* | 1a | M&O | 1a | M&O |
| *Pseudotriloculina* sp. 1 | 1a | M&O | 3 | M |
| *Pullenia quadriloba* | 2b | / |  |  |
| *Pyrgo elongata* |  |  | 3 | M |
| *Quinqueloculina auberiana* | 2b | / |  |  |
| *Quinquelcoculina berthelotiana* | 2a | M |  |  |
| *Quinqueloculina bicarinata* | 3 | M&O |  |  |
| *Quinqueloculina bosciana* | 2a | M&O |  |  |
| *Quinqueloculina contorta* | 3 | M |  |  |
| *Quinqueloculina* cf. *Q. irregularis* | 2b | / |  |  |
| *Quinqueloculina jugosa* |  |  | 3* | M&O |
| *Quinqueloculina* cf. *Q. laevigata* | 2a* | M&O | 3* | M&O |
| *Quinqueloculina limbata* | 3* | M&O | 1a | M&O |
| *Quinqueloculina parvula* | 2b | / | 2a | M&O |
| *Quinqueloculina seminula* | 1a | M&O | 1a | M&O |
| *Quinqueloculina stelligera* | 2a | M&O | 2b | / |
| *Quinqueloculina viennensis* | 3* | M&O |  |  |
| *Quinqueloculina vulgaris* | 2a | M&O |  |  |
| *Quinqueloculina* sp. 1 | 2b | / |  |  |
| *Quinqueloculina* sp. 4 | 2a | M |  |  |
| *Reophax* sp. 1 | 3* | M&O | 3 | M&O |
| *Reussella spinulosa* | 2b | / |  |  |
| *Rosalina bradyi* | 1a | M | 1b | / |
| *Rosalina bulloides* | 1a | M&O | 2a* | M&O |
| *Rosalina floridensis* | 1a | M | 3 | M |
| *Rosalina macropora* | 1a | M | 2b | / |
| *Rosalina* ? cf. *R. suezensis* | 2b | / |  |  |
| *Sigmoilinita costata* | 2b | / |  |  |
| *Siphonaperta dilatata* | 1a | M |  |  |
| *Siphonina reticulata* | 1a | M | 1b | / |
| *Sorites orbiculus* | 2b | / |  |  |
| *Sphaerogypsina* ? sp. 1 | 2b | / | 2b | / |
| *Spiroloculina angulosa* | 2b | / | 3 | O |
| *Spiroloculina antillarum* | 3 | M |  |  |
| *Spiroloculina cymbium* | 3 | M |  |  |
| *Spiroloculina krumbachi* | 2b | / |  |  |
| *Spiroloculina nitida* | 2a | M |  |  |
| *Spiroloculina ornata* | 2b | / |  |  |
| *Stomatorbina concentrica* | 3 | M |  |  |
| *Textularia bocki* | 2a* | M&O | 3* | M&O |
| *Textularia porrecta* | 3* | M&O | 3* | M&O |
| *Textularia* ? *truncata* | 3 | M |  |  |
| *Triloculina adriatica* | 2b | / |  |  |
| *Triloculina* cf. *T. fichteliana* | 2b | / |  |  |
| *Triloculina plicata* | 2b | / |  |  |
| *Triloculina schreiberiana* | 2a* | M&O | 3* | M&O |
| *Triloculina tricarinata* | 2b | / |  |  |
| *Trochammina inflata* | 2a* | M&O | 1a | M&O |
| *Uvigerina mediterranea* | 1a | M | 1b | / |
| *Valvulineria* sp. 1 | 2b | / | 3 | M |
| *Vertebralina striata* | 2a | M&O |  |  |
