# Supplementary material for: Effects of sampling site, season, and substrate on foraminiferal assemblages grown from propagule banks from lagoon sediments of Corfu Island (Greece, Ionian Sea)
Source: PLoS One. 2019 Jun 28;14(6):e0219015. doi: 10.1371/journal.pone.0219015 (PMC6599131; doi:10.1371/journal.pone.0219015)
Supplement: S5 Table — (DOCX) [file pone.0219015.s005.docx]

| **Species** | **“Functional groups”**  *modified from Langer (1993)*  species groups marked with an * show more than one life mode, but the generally most common has been used (see footnotes for further information) | **Foram-AMBI Group**  *from Jorissen et al (2018)*  1 = sensitive  2 = indifferent  3 = 3^rd^ order opportunists  4 = 2^nd^ order opportunists  5 = 1^st^ order opportunists  ua = unassigned |
| --- | --- | --- |
| *Adelosina carinatastriata* | epiphytic d | 1 |
| *Adelosina cliarensis* | epiphytic d | 1 |
| *Adelosina striata* | epiphytic d | ua |
| *Ammobaculites* sp. 1 | infaunal | ua |
| *Ammonia beccarii* | epifaunal*^1)^ | 2 |
| *Ammonia inflata* | epifaunal*^1)^ | 2 |
| *Ammonia parkinsoniana* | epifaunal*^1)^ | 1 |
| *Ammonia tepida* | epifaunal*^1)^ | 4 |
| *Ammonia* sp. 1 | epifaunal*^1)^ | ua |
| *Amphistegina lobifera* | epiphytic d | ua |
| *Asterigerinata mamilla* | epiphytic b | 1 |
| *Astrononion stelligerum* | epifaunal | 1 |
| *Aubignyna planidorso* | epifaunal*^2)^ | ua |
| *Bolivina pseudoplicata* | infaunal | 2 |
| *Bolivina variabilis* | infaunal | 2 |
| *Brizalina difformis* | infaunal | ua |
| *Brizalina* cf. *B. simpsoni* | infaunal | ua |
| *Brizalina spathulata* | infaunal | ua |
| *Brizalina striatula* | infaunal | 3 |
| *Brizalina* ? sp. 1 | infaunal | ua |
| *Buccella* sp. 1 | epifaunal*^2)^ | ua |
| *Bulimina costata* | infaunal | 2 |
| *Bulimina elongata* | infaunal | 3 |
| *Bulimina* cf. *B. marginata* | infaunal | 3 |
| *Cancris auriculus* | epifaunal | ua |
| *Cibicidella variabilis* | epiphytic b | 1 |
| *Cibicides advenum* | epiphytic b | ua |
| *Cibicides refulgens* | epiphytic b | 1 |
| *Clavulina angularis* | infaunal | ua |
| *Conorbella patelliformis* | epiphytic b | ua |
| *Cornuspira foliacea* | epiphytic b | ua |
| *Cycloforina contorta* | epiphytic d | 1 |
| *Cymbaloporetta plana* | epiphytic b | ua |
| *Cymbaloporetta squammosa* | epiphytic b | ua |
| *Dentalina* ? sp. 1 | infaunal | ua |
| *Dentalinoides* ? sp. 1 | infaunal | ua |
| *Disconorbis bulbosus* | infaunal | ua |
| *Discorbinella bertheloti* | epiphytic b | 1 |
| *Eggerelloides* sp. 1 | epiphytic d | ua |
| *Elphidium aculeatum* | epiphytic c | 1 |
| *Elphidium* cf. *E. advenum* | epiphytic c | 2 |
| *Elphidium crispum* | epiphytic c | 1 |
| *Elphidium depressulum* | epiphytic c | 2 |
| *Elphidium jenseni* | epiphytic c | ua |
| *Elphidium* cf. *E. jenseni* | epiphytic c | ua |
| *Elphidium macellum* | epiphytic c | 1 |
| *Elphidium williamsoni* | infaunal*^3)^ | ua |
| *Elphidium* sp. 1 | epiphytic c | ua |
| *Elphidium* sp. 2 | epiphytic c | ua |
| *Eponides concameratus* | epiphytic d | 1 |
| *Favulina* sp. 1 | infaunal | ua |
| *Floresina* sp. 1 | epifaunal | ua |
| *Fursenkoina* sp. 1 | infaunal | ua |
| *Gyroidinoides lamarckiana* | epifaunal | ua |
| *Haplophragmoides canariensis* | epifaunal | ua |
| *Haynesina depressula* | epifaunal*^4)^ | 2 |
| *Haynesina* sp. 1 | epifaunal*^4)^ | ua |
| *Heterolepa* cf. *H. subhaidingeri* | epifaunal | ua |
| *Hoeglundina elegans* | infaunal | ua |
| *Labrospira subglobosa* | infaunal | ua |
| *Laevipeneroplis karreri* | epiphytic d | 2 |
| *Lenticulina gibba* | infaunal | ua |
| *Lenticulina orbicularis* | infaunal | ua |
| *Lobatula lobatula* | infaunal | 1 |
| *Massilina gualtieriana* | epiphytic d | ua |
| *Massilina secans* | epiphytic d | 2 |
| *Melonis pompilioides* | infaunal | ua |
| *Miliammina fusca* | epifaunal | ua |
| *Miliolinella elongata* | epiphytic d | ua |
| *Miliolinella subrotunda* | epiphytic d | 1 |
| *Neoconorbina terquemi* | epiphytic b | 1 |
| *Nonionoides grateloupii* | infaunal | 2 |
| *Paracibicides* sp. 1 | epiphytic b | ua |
| *Parrina bradyi* | epiphytic d | ua |
| *Peneroplis pertusus* | epiphytic d | 1 |
| *Peneroplis planatus* | epiphytic d | 1 |
| *Planorbulina mediterranensis* | epiphytic a | 1 |
| *Planulina ariminensis* | epifaunal | ua |
| *Polymorphina* sp. 2 | infaunal | ua |
| *Polymorphina* sp. 3 | infaunal | ua |
| *Poroeponides* ? sp. 1 | epifaunal | ua |
| *Porosononion granosum* | epiphytic c | 3 |
| *Porosononion* sp. 1 | epiphytic c | ua |
| *Protoglobobulimina pupoides* | infaunal | ua |
| *Pseudoschlumbergerina ovata* | epiphytic d | ua |
| *Pseudotriloculina jugosa* | epiphytic d | ua |
| *Pseudotriloculina laevigata* | epiphytic d | 2 |
| *Pseudotriloculina* cf. *P. oblonga* | epiphytic d*^5)^ | ua |
| *Pseudotriloculina rotunda* | epiphytic d*^5)^ | ua |
| *Pseudotriloculina* sp. 1 | epiphytic d | ua |
| *Pullenia quadriloba* | infaunal | ua |
| *Pyrgo elongata* | epiphytic d | ua |
| *Quinqueloculina auberiana* | epiphytic d | 1 |
| *Quinquelcoculina berthelotiana* | epiphytic d | ua |
| *Quinqueloculina bicarinata* | epiphytic d | ua |
| *Quinqueloculina bosciana* | epiphytic d | 2 |
| *Quinqueloculina contorta* | epiphytic d | 1 |
| *Quinqueloculina* cf. *Q. irregularis* | epiphytic d | ua |
| *Quinqueloculina jugosa* | epiphytic d | ua |
| *Quinqueloculina* cf. *Q. laevigata* | epiphytic d | 1 |
| *Quinqueloculina limbata* | epiphytic d | ua |
| *Quinqueloculina parvula* | epiphytic d | 2 |
| *Quinqueloculina seminula* | epifaunal*^6)^ | 3 |
| *Quinqueloculina stelligera* | epiphytic d | 3 |
| *Quinqueloculina viennensis* | epiphytic d | 2 |
| *Quinqueloculina vulgaris* | epiphytic d | 1 |
| *Quinqueloculina* sp. 1 | epiphytic d | ua |
| *Quinqueloculina* sp. 4 | epiphytic d | ua |
| *Reophax* sp. 1 | infaunal | ua |
| *Reussella spinulosa* | infaunal | 1 |
| *Rosalina bradyi* | epiphytic b | 1 |
| *Rosalina bulloides* | epiphytic b | ua |
| *Rosalina floridensis* | epiphytic b | 1 |
| *Rosalina macropora* | epiphytic b | 1 |
| *Rosalina* ? cf. *R. suezensis* | epiphytic b | ua |
| *Sigmoilinita costata* | epiphytic d | 1 |
| *Siphonaperta dilatata* | epiphytic d | ua |
| *Siphonina reticulata* | epifaunal | ua |
| *Sorites orbiculus* | epiphytic c | 1 |
| *Sphaerogypsina* ? sp. 1 | epifaunal | ua |
| *Spiroloculina angulosa* | epiphytic d | ua |
| *Spiroloculina antillarum* | epiphytic d | 2 |
| *Spiroloculina cymbium* | epiphytic d | ua |
| *Spiroloculina krumbachi* | epiphytic d | ua |
| *Spiroloculina nitida* | epiphytic d | ua |
| *Spiroloculina ornata* | epiphytic d | ua |
| *Stomatorbina concentrica* | epifaunal | ua |
| *Textularia bocki* | epiphytic d | 3 |
| *Textularia porrecta* | epifaunal | ua |
| *Textularia* ? *truncata* | epifaunal | ua |
| *Triloculina adriatica* | epiphytic d | ua |
| *Triloculina* cf. *T. fichteliana* | epiphytic d | ua |
| *Triloculina plicata* | epiphytic d | 3 |
| *Triloculina schreiberiana* | epiphytic d | 3 |
| *Triloculina tricarinata* | epiphytic d | 1 |
| *Trochammina inflata* | epifaunal | ua |
| *Uvigerina mediterranea* | infaunal | 2 |
| *Valvulineria* sp. 1 | infaunal | ua |
| *Vertebralina striata* | epiphytic d | 1 |

^1)^ Also infaunal; especially *Ammonia tepida* has been described as epipelic (epifaunal) to endopelic (infaunal, Debenay et al 1998) and as sandy bottom species in sheltered zones (Aiello et al 2006)

^2)^ Also shallow infaunal

^3)^ As an unkeeled form of *Elphidium* infaunal in intertidal and subtidal areas (Murray 2006)

^4)^ Also infaunal, sandy bottom species in sheltered zones (Aiello et al 2006)

^5)^ Also epifaunal on sediment

^6)^ Also epiphytic d (permanently mobile on phytal and hard-ground substrates) and shallow infaunal, commonly occurring in the topmost centimetre of sediment (Barmawidjaja et al 1992)

Aiello, G.; Barra, D.; Coppa, M. G.; Valente, A.; Zeni, F. (2006) Recent infralittoral Foraminiferida and Ostracoda from Porto Cesareo Lagoon (Ionian Sea, Mediterranean). Bollettino della Società Paleontologica Italiana 45: 1-14.

Barmawidjaja, D. M.; Jorissen, Frans J.; Puskaric, S.; van der Zwaan, G.J (1992) Microhabitat selection by benthic foraminifera in the northern Adriatic Sea. Journal of Foraminiferal Research 22: 297-317.

Debenay, J.-P.; Bénéteau, E.; Zhang, J.; Stouff, V.; Geslin, E.; Redois, F.; Fernandez-Gonzalez, M. (1998) *Ammonia beccarii* and *Ammonia tepida* (Foraminifera): morphofunctional arguments for their distinction. Marine Micropaleontology 34: 235-244

Jorissen, F.; Nardelli, M.P.; Almogi-Labin, A.; Barras, C.; Bergamin, L.; Bicchi, E.; El Kateb, A.; Ferraro, L.; McGann, M.; Morigi, C.; Romano, E.; Sabbatini, A.; Schweizer, M.; Spezzaferri, S. (2018) Developing Foram-AMBI for biomonitoring in the Mediterranean: Species assignments to ecological categories. Marine Micropaleontology 140: 33-45

Langer, M.R. (1993) Epiphytic Foraminifera. Marine Micropaleontology 20: 235-265

Murray, J. (2006) Ecology and Applications of Benthic Foraminifera. Cambridge University Press, UK. 426 p.
