# Supplementary material for: Effects of sampling site, season, and substrate on foraminiferal assemblages grown from propagule banks from lagoon sediments of Corfu Island (Greece, Ionian Sea)
Source: PLoS One. 2019 Jun 28;14(6):e0219015. doi: 10.1371/journal.pone.0219015 (PMC6599131; doi:10.1371/journal.pone.0219015)
Supplement: S6 Table — (DOCX) [file pone.0219015.s006.docx]

| **May 17** | **30.05** | **02.06** | **06.06** | **09.06** | **14.06** | **19.06** | **23.06** | **27.06** | **30.06** | **04.07** | **07.07** | **11.07** |
| --- | --- | --- | --- | --- | --- | --- | --- | --- | --- | --- | --- | --- |
| Coral Pro | **Salinity** 40 ppt  **pH** ~8  **O_2_** 244 μmol/kg |  |  |  |  |  |  |  |  |  |  |  |
| 16 M.a |  | 40 ppt | 41 ppt | 40 ppt | 40 ppt | 40 ppt  pH ~8 O_2_ 213 | 40.5 | 40 ppt | 40.5 | 40 ppt | 40 ppt | 40 ppt  pH ~8 O_2_ 213 |
| 16 M.b |  | 40 ppt | 42 ppt | 40 ppt | 40 ppt | 40 ppt  pH ~8 O_2_ 244 | 40 ppt | 40 ppt | 40.5 | 40 ppt | 40 ppt | 40 ppt  pH ~8 O_2_ 183 |
| 16 P.a |  | 40 ppt | 40 ppt | 40 ppt | 40.5 | 41 ppt  pH ~8 O_2_ 213 | 40 ppt | 40 ppt | 40.5 | 40.5 | 40 ppt | 40 ppt  pH ~8 O_2_ 244 |
| 16 P.b |  | 40 ppt | 41 ppt | 40 ppt | 40 ppt | 40 ppt  pH ~8 O_2_ 213 | 40.5 | 40.5 | 41 ppt | 40 ppt | 40 ppt | 40 ppt  pH ~8 O_2_ 244 |
| 16 R.a |  | 40 ppt | 40 ppt | 41 ppt | 40 ppt | 40 ppt  pH ~8 O_2_ 213 | 40 ppt | 40 ppt | 40.5 | 40.5 | 40 ppt | 40 ppt  pH ~8 O_2_ 244 |
| 16 R.b |  | 40 ppt | 40 ppt | 41 ppt | 40 ppt | 40 ppt  pH ~8 O_2_ 213 | 40 ppt | 41 ppt | 40 ppt | 40 ppt | 40 ppt | 40 ppt  pH ~8 O_2_ 213 |
| 17 M.a |  | 40 ppt | 40 ppt | 40 ppt | 40 ppt | 40.5  pH ~8 O_2_ 213 | 40.5 | 40 ppt | 40 ppt | 40 ppt | 40 ppt | 40.5  pH ~8 O_2_ 213 |
| 17 M.b |  | 40 ppt | 40 ppt | 40 ppt | 40.5 | 40.5  pH ~8 O_2_ 213 | 40.5 | 40 ppt | 41 ppt | 40 ppt | 40 ppt | 40 ppt  pH ~8 O_2_ 213 |
| 17 P.a |  | 40 ppt | 40 ppt | 40 ppt | 40 ppt | 41 ppt  pH ~8 O_2_ 244 | 40 ppt | 40 ppt | 40 ppt | 40 ppt | 40.5 | 40.5  pH ~8 O_2_ 244 |
| 17 P.b |  | 40 ppt | 40 ppt | 40 ppt | 40 ppt | 41 ppt  pH ~8 O_2_ 244 | 40.5 | 40 ppt | 41 ppt | 40 ppt | 40 ppt | 40 ppt  pH ~8 O_2_ 213 |
| 17 R.a |  | 40 ppt | 40 ppt | 40 ppt | 40 ppt | 41 ppt  pH ~8 O_2_ 274 | 40 ppt | 40 ppt | 40 ppt | 40 ppt | 40 ppt | 40 ppt  pH ~8 O_2_ 213 |
| 17 R.b |  | 40 ppt | 40 ppt | 40 ppt | 40 ppt | 41 ppt  pH ~8 O_2_ 244 | 40 ppt | 40 ppt | 40.5 | 40.5 | 40 ppt | 41 ppt  pH ~8 O_2_ 244 |
| 19 M.a |  | 40 ppt | 40 ppt | 40 ppt | 41 ppt | 40 ppt  pH ~8 O_2_ 244 | 40.5 | 40.5 | 40 ppt | 40 ppt | 41 ppt | 40 ppt  pH ~8 O_2_ 183 |
| 19 M.b |  | 40 ppt | 40 ppt | 40 ppt | 41 ppt | 40 ppt  pH ~8 O_2_ 213 | 40.5 | 40 ppt | 40 ppt | 40 ppt | 40 ppt | 40 ppt  pH ~8 O_2_ 213 |
| 19 P.a |  | 40 ppt | 40 ppt | 40 ppt | 41 ppt | 40 ppt  pH ~8 O_2_ 213 | 40 ppt | 40 ppt | 40 ppt | 41 ppt | 40 ppt | 40 ppt  pH ~8 O_2_ 213 |
| 19 P.b |  | 40 ppt | 40 ppt | 40 ppt | 41 ppt | 40 ppt  pH ~8 O_2_ 244 | 40 ppt | 40 ppt | 40 ppt | 41 ppt | 40 ppt | 40.5  pH ~8 O_2_ 213 |
| 19 R.a |  | 40 ppt | 40 ppt | 40 ppt | 41 ppt | 40 ppt  pH ~8 O_2_ 244 | 40 ppt | 40 ppt | 40 ppt | 40 ppt | 40 ppt | 40.5  pH ~8 O_2_ 244 |
| 19 R.b |  | 40 ppt | 40 ppt | 40 ppt | 41 ppt | 40 ppt  pH ~8 O_2_ 244 | 40 ppt | 40 ppt | 40 ppt | 40.5 | 40 ppt | 40.5  pH ~8 O_2_ 244 |
| 20 M.a |  | 40 ppt | 40 ppt | 40 ppt | 40.5 | 41 ppt  pH ~8 O_2_ 244 | 40 ppt | 40 ppt | 40 ppt | 40 ppt | 40 ppt | 40.5  pH ~8 O_2_ 213 |
| 20 M.b |  | 40 ppt | 40 ppt | 40 ppt | 39 | 41 ppt  pH ~8 O_2_ 244 | 40 ppt | 40 ppt | 40 ppt | 40 ppt | 40 ppt | 40 ppt  pH ~8 O_2_ 213 |
| 20 P.a |  | 40 ppt | 40 ppt | 40 ppt | 40.5 | 40.5  pH ~8 O_2_ 213 | 40 ppt | 40.5 | 40 ppt | 40.5 | 40 ppt | 40 ppt  pH ~8 O_2_ 213 |
| 20 P.b |  | 40 ppt | 40 ppt | 40 ppt | 40 ppt | 41 ppt  pH ~8 O_2_ 244 | 40 ppt | 40 ppt | 40 ppt | 40.5 | 40 ppt | 40.5  pH ~8 O_2_ 244 |
| 20 R.a |  | 40 ppt | 40 ppt | 40 ppt | 41 ppt | 40 ppt  pH ~8 O_2_ 244 | 40 ppt | 40 ppt | 40 ppt | 41 ppt | 40 ppt | 40 ppt  pH ~8 O_2_ 213 |
| 20 R.b |  | 40 ppt | 41 ppt | 40 ppt | 40 ppt | 41 ppt  pH ~8 O_2_ 213 | 40.5 | 40 ppt | 40.5 | 40 ppt | 40 ppt | 41 ppt  pH ~8 O_2_ 244 |

| **Oct 17** | **17.10** | **19.10** | **23.10** | **26.10** | **30.10** | **02.11** | **06.11** | **09.11** | **13.11** | **16.11** | **20.11** | **23.11** | **27.11.** |
| --- | --- | --- | --- | --- | --- | --- | --- | --- | --- | --- | --- | --- | --- |
| Coral Pro | 40 ppt  pH ~8  O_2_ 7 mg/l |  |  |  |  |  |  |  |  |  |  |  |  |
| 59 M.a |  | 40 ppt | 40 ppt | 40 ppt | 40.5 | 40.5 | 40 ppt  pH ~8 O_2_ 5-6 | 40.5 | 40 ppt | 40 ppt | 40 ppt | 40.5 | 40.5  pH ~8 O_2_ 5 |
| 59 M.b |  | 40 ppt | 40 ppt | 40 ppt | 40 ppt | 40 ppt | 40 ppt  pH ~8 O_2_ 8 | 40 ppt | 40.5 | 40.5 | 40 ppt | 40.5 | 40.5  pH ~8 O_2_ 7 |
| 59 P.a |  | 39.5 | 40.5 | 40 ppt | 40.5 | 40.5 | 40 ppt  pH ~8 O_2_ 5-6 | 40.5 | 40 ppt | 40 ppt | 40 ppt | 40 ppt | 40.5  pH ~8 O_2_ 8 |
| 59 P.b |  | 39.5 | 40 ppt | 40 ppt | 40.5 | 40.5 | 40 ppt  pH ~8 O_2_ 6 | 40.5 | 40.5 | 40 ppt | 40 ppt | 40 ppt | 40.5  pH ~8 O_2_ 7 |
| 59 R.a |  | 39.5 | 40 ppt | 40 ppt | 40.5 | 40 ppt | 40 ppt  pH ~8 O_2_ 6 | 40.5 | 41 ppt | 40 ppt | 40 ppt | 40 ppt | 40 ppt  pH ~8 O_2_ 8 |
| 59 R.b |  | 39.5 | 40 ppt | 40 ppt | 40.5 | 40 ppt | 40 ppt  pH ~8 O_2_ 5-6 | 40.5 | 41 ppt | 40 ppt | 40 ppt | 40 ppt | 40.5  pH ~8 O_2_ 8 |
| 60 M.a |  | 40 ppt | 40 ppt | 40 ppt | 40 ppt | 40.5 | 40 ppt  pH ~8 O_2_ 5 | 40 ppt | 40 ppt | 40 ppt | 40.5 | 40 ppt | 40 ppt  pH ~8 O_2_ 7 |
| 60 M.b |  | 40 ppt | 40 ppt | 40 ppt | 40.5 | 40 ppt | 40 ppt  pH ~8 O_2_ 6 | 40 ppt | 40 ppt | 40 ppt | 40 ppt | 40 ppt | 40.5  pH ~8 O_2_ 8 |
| 60 P.a |  | 39.5 | 40 ppt | 40 ppt | 40.5 | 40.5 | 40.5  pH ~8 O_2_ 6 | 40 ppt | 40 ppt | 40 ppt | 40.5 | 40 ppt | 40.5  pH ~8 O_2_ 6 |
| 60 P.b |  | 39.5 | 40 ppt | 40 ppt | 40.5 | 40.5 | 40 ppt  pH ~8 O_2_ 6 | 40 ppt | 40 ppt | 40 ppt | 40 ppt | 40.5 | 40 ppt  pH ~8 O_2_ 7 |
| 60 R.a |  | 40 ppt | 40 ppt | 40 ppt | 40 ppt | 40.5 | 40 ppt  pH ~8 O_2_ 6 | 40 ppt | 40 ppt | 40 ppt | 40 ppt | 40 ppt | 41 ppt  pH ~8 O_2_ 7 |
| 60 R.b |  | 40 ppt | 40 ppt | 40 ppt | 41 ppt | 40 ppt | 40 ppt  pH ~8 O_2_ 6-7 | 40 ppt | 40.5 | 40 ppt | 40 ppt | 40 ppt | 40 ppt  pH ~8 O_2_ 7 |
| 61 M.a |  | 39 | 39 | 40 ppt | 40 ppt | 40.5 | 40 ppt  pH ~8 O_2_ 7 | 40 ppt | 40 ppt | 40 ppt | 40.5 | 40 ppt | 41 ppt  pH ~8 O_2_ 6 |
| 61 M.b |  | 38 | 40 ppt | 40 ppt | 40 ppt | 40 ppt | 40 ppt  pH ~8 O_2_ 7-8 | 40 ppt | 41 ppt | 40 ppt | 40 ppt | 40 ppt | 40.5  pH ~8 O_2_ ~8 |
| 61 P.a |  | 38 | 39.5 | 40 ppt | 40 ppt | 40.5 | 40 ppt  pH ~8 O_2_ 7 | 40 ppt | 40 ppt | 40 ppt | 40 ppt | 40.5 | 40 ppt  pH ~8 O_2_ 6 |
| 61 P.b |  | 37 | 40.5 | 40 ppt | 40 ppt | 40.5 | 40 ppt  pH ~8 O_2_ 7-8 | 40 ppt | 40.5 | 40 ppt | 40 ppt | 40 ppt | 40.5  pH ~8 O_2_ 8 |
| 61 R.a |  | 37 | 40 ppt | 40 ppt | 40 ppt | 40.5 | 40 ppt  pH ~8 O_2_ 7-8 | 40 ppt | 41 ppt | 40 ppt | 40 ppt | 40 ppt | 40.5  pH ~8 O_2_ 7 |
| 61 R.b |  | 38 | 40 ppt | 40 ppt | 40 ppt | 40 ppt | 40 ppt  pH ~8 O_2_ 7 | 40 ppt | 40 ppt | 40 ppt | 40 ppt | 40 ppt | 40.5  pH ~8 O_2_ 5 |
| 62 M.a |  | 38 | 40 ppt | 40 ppt | 40 ppt | 40 ppt | 40 ppt  pH ~8 O_2_ 7-8 | 40 ppt | 40 ppt | 40.5 | 40 ppt | 40.5 | 41 ppt  ph ~8 O_2_ 7 |
| 62 M.b |  | 38 | 40 ppt | 40 ppt | 40 ppt | 40 ppt | 40 ppt  pH ~8 O_2_ 7 | 40 ppt | 40 ppt | 40 ppt | 40 ppt | 41 ppt | 41 ppt  pH ~8 O_2_ 8 |
| 62 P.a |  | 37 | 40 ppt | 40.5 | 40 ppt | 40 ppt | 40.5  pH ~8 O_2_ 8 | 40.5 | 40.5 | 40.5 | 40 ppt | 41 ppt | 40 ppt  pH ~8 O_2_ 6 |
| 62 P.b |  | 38 | 40 ppt | 40 ppt | 40 ppt | 40 ppt | 40 ppt  pH ~8 O_2_ 8 | 40.5 | 40.5 | 40 ppt | 40 ppt | 41 ppt | 40.5  pH ~8 O_2_ 8 |
| 62 R.a |  | 38 | 41 ppt | 40 ppt | 40 ppt | 40 ppt | 40.5  pH ~8 O_2_ 7-8 | 40.5 | 40 ppt | 40.5 | 40 ppt | 40.5 | 40.5  pH ~8 O_2_ 8 |
| 62 R.b |  | 37 | 40 ppt | 40 ppt | 40.5 | 40 ppt | 40 ppt  pH ~8 O_2_ 7-8 | 40.5 | 40 ppt | 40 ppt | 40 ppt | 40 ppt | 40 ppt  pH ~8 O_2_ 7-8 |
