# Supplementary material for: Effects of sampling site, season, and substrate on foraminiferal assemblages grown from propagule banks from lagoon sediments of Corfu Island (Greece, Ionian Sea)
Source: PLoS One. 2019 Jun 28;14(6):e0219015. doi: 10.1371/journal.pone.0219015 (PMC6599131; doi:10.1371/journal.pone.0219015)
Supplement: S7 Table — (DOCX) [file pone.0219015.s007.docx]

**Family Rzehakinidae Cushman, 1933**

*Miliammina fusca* (Brady, 1870)

**Family Hormosinidae Haeckel, 1894**

*Reophax* sp. 1

**Family Haplophragmoididae Maync, 1952**

*Haplophragmoides canariensis* (d’Orbigny, 1839)

*Labrospira subglobosa* (Cushman, 1910)

**Family Lituolidae de Blainville, 1827**

*Ammobaculites* sp. 1

**Family Trochamminidae Schwager, 1877**

*Trochammina inflata* (Montagu, 1808)

**Family Eggerellidae Schwager, 1877**

*Eggerelloides* sp. 1

**Family Textulariidae Ehrenberg, 1838**

*Textularia bocki* Höglund, 1947

*Textularia porrecta* Brady, 1884

*Textularia* ? *truncata* Höglund, 1947

**Family Valvulinidae Berthelin, 1880**

*Clavulina angularis* d’Orbigny, 1826

**Family Cornuspiridae Schultze, 1854**

*Cornuspira foliacea* (Philippi, 1844)

**Family Fischerinidae Millett, 1898**

*Vertebralina striata* d’Orbigny, 1826

**Family Spiroloculinidae Wiesner, 1920**

*Adelosina carinatastriata* (Wiesner, 1923)

*Adelosina cliarensis* (Heron-Allen & Earland, 1930)

*Adelosina striata* d’Orbigny, 1826

*Spiroloculina angulosa* d’Orbigny in Fornasini, 1904

*Spiroloculina antillarum* d’Orbigny, 1839

*Spiroloculina cymbium* d’Orbigny, 1839

*Spiroloculina krumbachi* Wiesner, 1911

*Spiroloculina nitida* d’Orbigny, 1826

*Spiroloculina ornata* d’Orbigny, 1839

**Family Hauerinidae Schwager, 1876**

*Siphonaperta dilatata* (Le Calvez & Le Calvez, 1958)

*Cycloforina contorta* (d’Orbigny, 1846)

*Massilina gualtieriana* (d’Orbigny, 1839)

*Massilina secans* (d’Orbigny, 1826)

*Quinqueloculina auberiana* d’Orbigny, 1839

*Quinqueloculina berthelotiana* d’Orbigny, 1839

*Quinqueloculina bicarinata* d’Orbigny in Terquem, 1878

*Quinqueloculina bosciana* d’Orbigny, 1839

*Quinqueloculina* *contorta* d’Orbigny, 1846

*Quinqueloculina* cf. *Q. irregularis* d’Orbigny in Terquem, 1878

*Quinqueloculina jugosa* Cushman, 1944

*Quinqueloculina* cf. *Q. laevigata* d’Orbigny, 1839

*Quinqueloculina limbata* d’Orbigny, 1826

*Quinqueloculina parvula* Schlumberger, 1894

*Quinqueloculina seminula* (Linnaeus, 1758)

*Quinqueloculina stelligera* Schlumberger, 1893

*Quinqueloculina viennensis* Le Calvez & Le Calvez, 1958

*Quinqueloculina vulgaris* d’Orbigny, 1826

*Quinqueloculina* sp. 1

*Quinqueloculina* sp. 4

*Miliolinella elongata* Kruit, 1955

*Miliolinella subrotunda* (Montagu, 1803)

*Pseudotriloculina jugosa* (Cushman, 1944)

*Pseudotriloculina laevigata* (d’Orbigny in Terquem, 1878)

*Pseudotriloculina* cf. *P. oblonga* (Montagu, 1803)

*Pseudotriloculina rotunda* (d’Orbigny in Schlumberger, 1893)

*Pseudotriloculina* sp. 1

*Pyrgo elongata* (d’Orbigny, 1826)

*Triloculina adriatica* Le Calvez & Le Calvez, 1958

*Triloculina* cf. *T. fichteliana* (d’Orbigny, 1839)

*Triloculina plicata* Terquem, 1878

*Triloculina schreiberiana* d’Orbigny, 1839

*Triloculina tricarinata* d’Orbigny in Deshayes, 1832

*Pseudoschlumbergerina ovata* (Sidebottom, 1904)

*Sigmoilinita costata* (Schlumberger, 1893)

*Parrina bradyi* (Millett, 1898)

**Family Peneroplidae Schultze, 1854**

*Laevipeneroplis karreri* (Wiesner, 1923)

*Peneroplis pertusus* (Forsskål in Niebuhr, 1775)

*Peneroplis planatus* (Fichtel & Moll, 1798)

**Family Soritidae Ehrenberg, 1839**

*Sorites orbiculus* (Forsskål in Niebuhr, 1775)

**Family Nodosariidae Ehrenberg, 1838**

*Dentalina* ? sp. 1

*Dentalinoides* ? sp. 1

**Family Vaginulinidae Reuss, 1860**

*Lenticulina gibba* (d’Orbigny, 1839)

*Lenticulina orbicularis* (d’Orbigny, 1826)

**Family Polymorphinidae d’Orbigny, 1839**

*Polymorphina* sp. 2

*Polymorphina* sp. 3

**Family Ellipsolagenidae Silvestri, 1923**

*Favulina* sp. 1

**Family Epistominidae Wedekind, 1937**

*Hoeglundina elegans* (d’Orbigny, 1826)

**Family Bolivinidae Glaessner, 1937**

*Bolivina pseudoplicata* Heron-Allen & Earland, 1930

*Bolivina variabilis* (Williamson, 1858)

*Brizalina difformis* (Williamson, 1848)

*Brizalina* cf. *B. simpsoni* (Heron-Allen & Earland, 1915)

*Brizalina spathulata* (Williamson, 1858)

*Brizalina striatula* (Cushman, 1922)

*Brizalina* ? sp. 1

**Family Turrilinidae Cushman, 1927**

*Floresina* sp. 1

**Family Buliminidae Jones, 1875**

*Bulimina costata* d’Orbigny, 1852

*Bulimina elongata* d’Orbigny, 1846

*Bulimina* cf*. B. marginata* d’Orbigny, 1826

*Protoglobobulimina pupoides* (d’Orbigny, 1846)

**Familiy Uvigerinidae Haeckel, 1894**

*Uvigerina mediterranea* Hofker, 1932

**Familiy Reussellidae Cushman, 1933**

*Reussella spinulosa* (Reuss, 1850)

**Familiy Fursenkoinidae Loeblich & Tappan, 1961**

*Fursenkoina* sp. 1

**Family Bagginidae Cushman, 1927**

*Cancris auriculus* (Fichtel & Moll, 1798)

*Valvulineria* sp. 1

**Family Eponididae Hofker, 1951**

*Eponides concameratus* (Montagu 1808)

*Poroeponides* ? sp. 1

**Family Mississippinidae Saidova, 1981**

*Stomatorbina concentrica* (Parker & Jones, 1864)

**Family Discorbidae Ehrenberg, 1838**

*Disconorbis bulbosus* (Parker, 1954)

**Family Rosalinidae Reiss, 1963**

*Neoconorbina terquemi* (Rzehak, 1888)

*Rosalina bradyi* Cushman, 1915

*Rosalina bulloides* d’Orbigny, 1839

*Rosalina floridensis* (Cushman, 1930)

*Rosalina macropora* (Hofker, 1951)

*Rosalina* ? cf. *R. suezensis* (Said, 1949)

**Family Glabratellidae Loeblich & Tappan, 1964**

*Conorbella patelliformis* (Brady, 1884)

**Family Siphoninidae Cushman, 1927**

*Siphonina reticulata* (Cžjžek, 1848)

**Family Discorbinellidae Sigal, 1952**

*Discorbinella bertheloti* (d’Orbigny, 1839)

**Family Planulinidae Bermúdez, 1952**

*Planulina ariminensis* d’Orbigny, 1826

**Family Cibicididae Cushman, 1927**

*Cibicides advenum* (d’Orbigny, 1839)

*Cibicides refulgens* Montford, 1808

*Lobatula lobatula* (Walker & Jacob, 1798)

*Paracibicides* sp. 1

**Family Planorbulinidae Schwager, 1877**

*Cibicidella variabilis* (d’Orbigny, 1826)

*Planorbulina mediterranensis* d’Orbigny, 1826

**Family Cymbaloporidae Cushman, 1927**

*Cymbaloporetta plana* (Cushman, 1924)

*Cymbaloporetta squammosa* (d’Orbigny, 1839)

**Family Acervulinidae Schultze, 1854**

*Sphaerogypsina* ? sp. 1

**Family Asterigerinatidae Reiss, 1963**

*Asterigerinata mamilla* (Williamson, 1858)

**Family Amphisteginidae Cushman, 1927**

*Amphistegina lobifera* Larsen, 1976

**Familiy Nonionidae Schultze, 1854**

*Haynesina depressula* (Walker & Jacob, 1798)

*Haynesina* sp. 1

*Nonionoides grateloupii* (d’Orbigny, 1839)

*Astrononion stelligerum* (d’Orbigny, 1839)

*Melonis pompilioides* (Fichtel & Moll, 1798)

*Pullenia quadriloba* Reuss, 1867

**Family Heterolepidae Gonzáles-Donoso, 1969**

*Heterolepa* cf. *H. subhaidingeri* (Parr, 1950)

**Family Gavelinellidae Hofker, 1956**

*Gyroidinoides lamarckiana* (d’Orbigny, 1839)

**Family Coleitidae Loeblich & Tappan, 1984**

*Aubignyna planidorso* (Atkinson, 1969)

*Buccella* sp. 1

**Family Rotaliidae Ehrenberg, 1839**

*Ammonia beccarii* (Linnaeus, 1758)

*Ammonia inflata* (Seguenza, 1862)

*Ammonia parkinsoniana* (d’Orbigny, 1839)

*Ammonia tepida* (Cushman, 1926)

*Ammonia* sp. 1

**Family Elphidiidae Galloway, 1933**

*Elphidium aculeatum* (d’Orbigny, 1846)

*Elphidium* cf. *E. advenum* (Cushman, 1922)

*Elphidium crispum* (Linnaeus, 1758)

*Elphidium depressulum* Cushman, 1933

*Elphidium jenseni* (Cushman, 1924)

*Elphidium* cf. *E. jenseni* (Cushman, 1924)

*Elphidium macellum* (Fichtel & Moll, 1798)

*Elphidium williamsoni* Haynes, 1973

*Elphidium* sp. 1

*Elphidium* sp. 2

*Porosononion granosum* (d’Orbigny, 1846)

*Porosononion* sp. 1
